# Supplementary material for: Findings of new phytoplankton species in the Barents Sea as a consequence of global climate changes
Source: PeerJ. 2023 Jun 13;11:e15472. doi: 10.7717/peerj.15472 (PMC10274593; doi:10.7717/peerj.15472)
Supplement: Supplemental Information 1 — All data are the results from research vessel –”Dalnie Zelentsy” (IMO Number 7740477) for the period between 2007-2019. [file peerj-11-15472-s001.docx]

**Table A1:**

**Location of sampling station.
All data are the results from research vessel – "Dalnie Zelentsy" (IMO Number 7740477) for period 2007-2019.**

| **ID** | **Date** | **Time (**UTC+3**)** | **Latitude**  **(dig. degree)** | **Longitude**  **(dig. degree)** | **Depth, m** |
| --- | --- | --- | --- | --- | --- |
|  | 19.08.2007 | 00:43 | 69.50033 | 33.50415 | 266 |
|  | 19.08.2007 | 10:14 | 69.99868 | 33.54838 | 150 |
|  | 19.08.2007 | 15:50 | 70.50217 | 33.49033 | 245 |
|  | 19.08.2007 | 21:40 | 71.00032 | 33.50390 | 221 |
|  | 20.08.2007 | 03:05 | 71.29943 | 33.49738 | 247 |
|  | 20.08.2007 | 05:06 | 71.49943 | 33.51013 | 273 |
|  | 20.08.2007 | 09:03 | 71.80272 | 33.50203 | 315 |
|  | 20.08.2007 | 12:10 | 72.00148 | 33.50443 | 264 |
|  | 20.08.2007 | 18:15 | 72.33508 | 33.49517 | 273 |
|  | 20.08.2007 | 20:48 | 72.50207 | 33.50092 | 280 |
|  | 21.08.2007 | 06:04 | 72.99917 | 33.51848 | 212 |
|  | 21.08.2007 | 13:48 | 73.50000 | 33.50873 | 279 |
|  | 22.08.2007 | 08:25 | 73.99950 | 33.50017 | 317 |
|  | 23.08.2007 | 00:45 | 74.50103 | 41.00023 | 206 |
|  | 23.08.2007 | 03:25 | 74.50000 | 39.80338 | 180 |
|  | 23.08.2007 | 08:30 | 74.49998 | 37.24458 | 159 |
|  | 23.08.2007 | 10:55 | 74.50040 | 36.42778 | 232 |
|  | 23.08.2007 | 12:46 | 74.50023 | 35.77333 | 260 |
|  | 23.08.2007 | 15:36 | 74.50003 | 35.00375 | 269 |
|  | 23.08.2007 | 18:52 | 74.50218 | 33.49810 | 257 |
|  | 23.08.2007 | 22:34 | 75.00435 | 33.50372 | 144 |
|  | 24.08.2007 | 02:20 | 75.50067 | 33.50018 | 222 |
|  | 24.08.2007 | 06:02 | 76.00335 | 33.50867 | 300 |
|  | 24.08.2007 | 10:05 | 76.49993 | 33.49610 | 209 |
|  | 24.08.2007 | 13:55 | 77.00028 | 33.49590 | 156 |
|  | 25.08.2007 | 13:25 | 78.99885 | 33.58167 | 230 |
|  | 25.08.2007 | 18:10 | 79.50147 | 34.99925 | 326 |
|  | 26.08.2007 | 01:25 | 80.00165 | 34.99000 | 94 |
|  | 26.08.2007 | 05:38 | 80.50017 | 34.99468 | 143 |
|  | 26.08.2007 | 10:03 | 81.00212 | 35.00282 | 175 |
|  | 26.08.2007 | 14:00 | 81.36500 | 35.00168 | 175 |
|  | 26.08.2007 | 20:10 | 80.96667 | 37.14220 | 101 |
|  | 26.08.2007 | 22:27 | 80.94632 | 38.93330 | 218 |
|  | 27.08.2007 | 12:12 | 80.93150 | 40.74333 | 570 |
|  | 27.08.2007 | 22:34 | 80.89585 | 42.80365 | 473 |
|  | 28.08.2007 | 05:37 | 80.88245 | 44.90963 | 258 |
|  | 28.08.2007 | 08:25 | 80.87452 | 46.15938 | 211 |
|  | 30.08.2007 | 16:26 | 80.33587 | 52.76972 | 12 |
|  | 31.08.2007 | 14:53 | 77.83347 | 56.87533 | 261 |
|  | 31.08.2007 | 17:05 | 77.63450 | 57.70270 | 174 |
|  | 31.08.2007 | 22:43 | 76.95128 | 59.71005 | 217 |
|  | 01.09.2007 | 13:46 | 75.53760 | 56.33925 | 161 |
|  | 01.09.2007 | 16:25 | 75.41478 | 56.00612 | 158 |
|  | 01.09.2007 | 20:06 | 75.23350 | 55.16923 | 205 |
|  | 02.09.2007 | 01:26 | 74.72500 | 54.89000 | 127 |
|  | 02.09.2007 | 06:25 | 74.20152 | 54.00455 | 161 |
|  | 02.09.2007 | 12:36 | 73.45377 | 53.00245 | 65 |
|  | 02.09.2007 | 19:25 | 72.84962 | 51.75172 | 83 |
|  | 02.09.2007 | 21:10 | 72.66655 | 51.58120 | 77 |
|  | 02.09.2007 | 23:50 | 72.59952 | 50.68190 | 160 |
|  | 03.09.2007 | 02:32 | 72.43333 | 49.73965 | 104 |
|  | 03.09.2007 | 06:30 | 72.06445 | 48.25482 | 203 |
|  | 03.09.2007 | 08:31 | 71.89910 | 47.86850 | 63 |
|  | 03.09.2007 | 14:45 | 71.60058 | 46.95417 | 53 |
|  | 03.09.2007 | 20:00 | 71.33385 | 45.56787 | 69 |
|  | 03.09.2007 | 23:58 | 71.14798 | 45.04775 | 257 |
|  | 04.09.2007 | 03:06 | 70.99928 | 44.31920 | 137 |
|  | 04.09.2007 | 06:42 | 70.88252 | 43.84633 | 120 |
|  | 04.09.2007 | 08:02 | 70.63472 | 42.95220 | 75 |
|  | 04.09.2007 | 13:15 | 70.49597 | 42.35667 | 62 |
|  | 04.09.2007 | 18:21 | 70.26390 | 41.74723 | 95 |
|  | 04.09.2007 | 19:42 | 70.18168 | 41.31525 | 193 |
|  | 04.09.2007 | 21:39 | 70.05003 | 40.72900 | 102 |
|  | 05.09.2007 | 00:32 | 69.85295 | 39.88667 | 225 |
|  | 05.09.2007 | 08:08 | 69.50145 | 38.55123 | 113 |
|  | 05.09.2007 | 10:13 | 69.26575 | 37.99627 | 221 |
|  | 05.09.2007 | 20:00 | 69.70210 | 34.03120 | 194 |
|  | 06.09.2007 | 08:11 | 69.64028 | 33.88315 | 253 |
|  | 06.09.2007 | 10:02 | 69.58540 | 33.75190 | 200 |
|  | 06.09.2007 | 12:50 | 69.50500 | 33.38242 | 214 |
|  | 06.09.2007 | 14:39 | 69.50000 | 33.50065 | 260 |
|  | 31.07.2007 | 22:30 | 69.39017 | 33.58667 | 190 |
|  | 01.08.2007 | 09:52 | 69.58792 | 33.59400 | 172 |
|  | 01.08.2007 | 16:40 | 69.55735 | 34.47868 | 152 |
|  | 04.08.2007 | 19:15 | 69.87323 | 33.47677 | 218 |
|  | 05.08.2007 | 09:57 | 69.53920 | 32.84777 | 261 |
|  | 01.06.2007 | 16:56 | 68.53147 | 38.74798 | 95 |
|  | 02.06.2007 | 20:28 | 68.84280 | 38.08372 | 141 |
|  | 02.06.2007 | 00:36 | 69.15348 | 36.89615 | 189 |
|  | 02.06.2007 | 03:43 | 69.32250 | 36.13600 | 175 |
|  | 02.06.2007 | 06:25 | 69.41892 | 35.31333 | 145 |
|  | 02.06.2007 | 10:40 | 69.55470 | 34.21257 | 237 |
|  | 02.06.2007 | 13:02 | 69.61433 | 33.51200 | 222 |
|  | 02.06.2007 | 14:18 | 69.49852 | 33.50133 | 259 |
|  | 30.09.2007 | 18:45 | 69.86657 | 33.47698 | 224 |
|  | 01.10.2007 | 10:34 | 69.55450 | 34.48697 | 152 |
|  | 01.10.2007 | 18:40 | 69.58868 | 33.60793 | 169 |
|  | 02.10.2007 | 08:51 | 69.39873 | 33.60732 | 181 |
|  | 02.10.2007 | 14:24 | 69.53783 | 32.85030 | 261 |
|  | 16.08.2008 | 22:20 | 69.50033 | 33.50083 | 259 |
|  | 17.08.2008 | 05:04 | 69.99983 | 33.49435 | 253 |
|  | 17.08.2008 | 09:40 | 70.50065 | 33.50740 | 253 |
|  | 17.08.2008 | 17:15 | 71.00105 | 33.49867 | 225 |
|  | 17.08.2008 | 20:05 | 71.29977 | 33.49858 | 252 |
|  | 17.08.2008 | 22:25 | 71.49577 | 33.50378 | 281 |
|  | 18.08.2008 | 05:05 | 71.79955 | 33.49963 | 325 |
|  | 18.08.2008 | 07:11 | 71.99982 | 33.50045 | 266 |
|  | 18.08.2008 | 10:30 | 72.33320 | 33.49513 | 285 |
|  | 18.08.2008 | 12:25 | 72.49953 | 33.50462 | 290 |
|  | 18.08.2008 | 20:50 | 72.99737 | 33.50077 | 215 |
|  | 19.08.2008 | 04:44 | 73.50000 | 33.48765 | 287 |
|  | 19.08.2008 | 13:25 | 74.00200 | 33.49735 | 322 |
|  | 19.08.2008 | 17:00 | 74.49980 | 33.49682 | 264 |
|  | 19.08.2008 | 23:10 | 75.01500 | 33.49012 | 142 |
|  | 20.08.2008 | 04:18 | 75.49890 | 33.48835 | 231 |
|  | 20.08.2008 | 10:25 | 76.00038 | 33.51693 | 307 |
|  | 20.08.2008 | 14:37 | 76.00007 | 31.32600 | 330 |
|  | 21.08.2008 | 00:04 | 76.09663 | 26.99172 | 210 |
|  | 21.08.2008 | 06:58 | 76.18403 | 23.19627 | 48.7 |
|  | 21.08.2008 | 15:05 | 76.26638 | 19.16773 | 266 |
|  | 21.08.2008 | 19:53 | 76.36690 | 16.39933 | 60.3 |
|  | 26.08.2008 | 06:14 | 76.50412 | 33.48770 | 211 |
|  | 26.08.2008 | 09:15 | 77.00408 | 33.48900 | 159 |
|  | 26.08.2008 | 16:35 | 77.50408 | 33.49122 | 155 |
|  | 26.08.2008 | 20:05 | 78.00375 | 33.48717 | 184 |
|  | 26.08.2008 | 23:55 | 78.50450 | 33.49558 | 231 |
|  | 27.08.2008 | 05:39 | 78.83620 | 37.33820 | 213 |
|  | 27.08.2008 | 13:10 | 79.20005 | 41.82908 | 356 |
|  | 27.08.2008 | 18:32 | 79.43657 | 44.99458 | 91.2 |
|  | 27.08.2008 | 23:10 | 79.67090 | 48.20908 | 307 |
|  | 28.08.2008 | 05:20 | 79.79355 | 53.08762 | 463 |
|  | 30.08.2008 | 01:33 | 79.68097 | 60.29752 | 168.5 |
|  | 30.08.2008 | 06:36 | 79.26337 | 61.67667 | 195 |
|  | 30.08.2008 | 12:07 | 78.82972 | 63.06867 | 322.6 |
|  | 30.08.2008 | 16:48 | 78.26457 | 64.78323 | 376.9 |
|  | 31.08.2008 | 02:01 | 77.83307 | 66.06155 | 327.7 |
|  | 31.08.2008 | 05:21 | 77.42822 | 67.19887 | 279.9 |
|  | 31.08.2008 | 07:18 | 77.20167 | 67.81918 | 272.2 |
|  | 31.08.2008 | 16:51 | 76.79933 | 64.76797 | 217 |
|  | 31.08.2008 | 03:25 | 76.36608 | 59.86402 | 122.6 |
|  | 31.08.2008 | 03:25 | 75.70248 | 57.10868 | 150 |
|  | 01.09.2008 | 13:14 | 75.53630 | 56.49987 | 154.6 |
|  | 01.09.2008 | 18:48 | 75.23643 | 55.17033 | 200.9 |
|  | 01.09.2008 | 22:45 | 74.72513 | 54.87135 | 132 |
|  | 02.09.2008 | 08:33 | 73.45117 | 52.97920 | 65 |
|  | 02.09.2008 | 13:30 | 72.84810 | 51.75100 | 84 |
|  | 02.09.2008 | 15:02 | 72.66705 | 51.57885 | 77 |
|  | 02.09.2008 | 19:16 | 72.59883 | 50.68753 | 164 |
|  | 02.09.2008 | 22:00 | 72.43822 | 49.72883 | 105 |
|  | 03.09.2008 | 11:10 | 71.58428 | 46.96510 | 52 |
|  | 04.09.2008 | 02:40 | 70.63470 | 42.95208 | 75 |
|  | 04.09.2008 | 04:31 | 70.44777 | 42.36538 | 62 |
|  | 04.09.2008 | 07:02 | 70.26475 | 41.75175 | 97 |
|  | 04.09.2008 | 09:10 | 70.18177 | 41.32883 | 180 |
|  | 04.09.2008 | 18:16 | 69.85208 | 39.88692 | 230 |
|  | 05.09.2008 | 01:07 | 69.50217 | 38.55150 | 115 |
|  | 05.09.2008 | 03:33 | 69.26578 | 37.99925 | 220 |
|  | 29.07.2008 | 17:52 | 67.61345 | 41.97813 | 36 |
|  | 29.07.2008 | 23:10 | 67.93948 | 40.91367 | 64 |
|  | 30.07.2008 | 03:18 | 68.21325 | 40.10780 | 72 |
|  | 30.07.2008 | 14:17 | 68.53158 | 38.74577 | 100 |
|  | 30.07.2008 | 18:42 | 68.83805 | 38.08912 | 149 |
|  | 30.07.2008 | 23:00 | 69.06207 | 36.71607 | 80 |
|  | 31.07.2008 | 03:53 | 69.32192 | 36.13962 | 186 |
|  | 31.07.2008 | 06:34 | 69.41735 | 35.32127 | 154 |
|  | 31.07.2008 | 10:15 | 69.55148 | 34.20775 | 245 |
|  | 31.07.2008 | 12:29 | 69.49895 | 33.50107 | 267 |
|  | 01.08.2009 | 09:35 | 71.43347 | 25.46702 | 293 |
|  | 01.08.2009 | 15:54 | 71.55140 | 25.02710 | 293 |
|  | 01.08.2009 | 19:08 | 71.79952 | 24.58425 | 284 |
|  | 01.08.2009 | 21:27 | 72.03448 | 24.05660 | 288 |
|  | 02.08.2009 | 01:17 | 72.25240 | 23.66348 | 283 |
|  | 02.08.2009 | 03:31 | 72.45165 | 23.19583 | 311 |
|  | 02.08.2009 | 08:48 | 72.71545 | 22.57612 | 391 |
|  | 02.08.2009 | 12:12 | 73.01638 | 21.99917 | 443 |
|  | 02.08.2009 | 15:43 | 73.28585 | 21.44663 | 450 |
|  | 02.08.2009 | 18:42 | 73.58345 | 20.76432 | 503 |
|  | 02.08.2009 | 22:53 | 73.73485 | 20.46318 | 463 |
|  | 03.08.2009 | 01:43 | 73.86995 | 20.13723 | 293 |
|  | 03.08.2009 | 03:16 | 74.01833 | 19.83167 | 122 |
|  | 03.08.2009 | 05:41 | 74.22885 | 19.33653 | 70.7 |
|  | 03.08.2009 | 12:29 | 74.60128 | 18.75230 | 63.5 |
|  | 03.08.2009 | 15:40 | 74.80457 | 18.48747 | 268 |
|  | 03.08.2009 | 18:26 | 75.13402 | 18.16727 | 71.5 |
|  | 03.08.2009 | 20:56 | 75.45055 | 17.80410 | 140 |
|  | 04.08.2009 | 01:38 | 75.76892 | 17.46297 | 249 |
|  | 04.08.2009 | 03:38 | 76.00285 | 17.16240 | 322 |
|  | 04.08.2009 | 08:20 | 76.24922 | 16.86573 | 219 |
|  | 04.08.2009 | 12:20 | 76.37270 | 16.70915 | 43 |
|  | 04.08.2009 | 16:27 | 76.75023 | 15.16890 | 60.1 |
|  | 04.08.2009 | 21:36 | 77.08268 | 12.90482 | 149 |
|  | 05.08.2009 | 03:05 | 77.41885 | 11.00057 | 930 |
|  | 05.08.2009 | 10:43 | 77.63302 | 11.63818 | 184 |
|  | 05.08.2009 | 13:05 | 77.83148 | 12.24548 | 44 |
|  | 08.08.2009 | 02:32 | 77.26635 | 23.63912 | 82 |
|  | 08.08.2009 | 10:12 | 77.75533 | 27.25938 | 122 |
|  | 08.08.2009 | 14:30 | 77.94095 | 28.61248 | 275 |
|  | 08.08.2009 | 18:33 | 78.13655 | 30.21022 | 311 |
|  | 09.08.2009 | 00:15 | 78.32857 | 31.87633 | 227 |
|  | 09.08.2009 | 03:08 | 78.49867 | 33.49847 | 243 |
|  | 09.08.2009 | 06:23 | 78.67225 | 35.45327 | 154 |
|  | 09.08.2009 | 09:42 | 78.83587 | 37.33502 | 216 |
|  | 09.08.2009 | 13:13 | 78.99847 | 39.32893 | 179 |
|  | 09.08.2009 | 18:16 | 79.11482 | 41.83197 | 385 |
|  | 09.08.2009 | 21:40 | 79.31602 | 43.52375 | 271 |
|  | 10.08.2009 | 00:10 | 79.43497 | 44.99477 | 91.5 |
|  | 10.08.2009 | 03:08 | 79.56172 | 46.71647 | 311 |
|  | 10.08.2009 | 07:18 | 79.67050 | 48.21058 | 318 |
|  | 10.08.2009 | 10:41 | 79.83860 | 50.34783 | 55 |
|  | 12.08.2009 | 21:26 | 77.99952 | 33.50055 | 190 |
|  | 13.08.2009 | 01:45 | 77.49875 | 33.50208 | 147 |
|  | 13.08.2009 | 05:25 | 76.99958 | 33.49987 | 162 |
|  | 13.08.2009 | 09:46 | 76.50020 | 33.50318 | 213 |
|  | 13.08.2009 | 13:29 | 76.00000 | 33.50138 | 310 |
|  | 13.08.2009 | 18:08 | 75.50008 | 33.49405 | 228 |
|  | 13.08.2009 | 22:20 | 74.99952 | 33.49995 | 147 |
|  | 14.08.2009 | 03:32 | 74.50002 | 33.49168 | 265 |
|  | 15.08.2009 | 15:19 | 73.99823 | 33.49557 | 329 |
|  | 15.08.2009 | 18:51 | 73.49758 | 33.49638 | 288 |
|  | 15.08.2009 | 23:03 | 72.99870 | 33.49852 | 216 |
|  | 16.08.2009 | 04:28 | 72.49880 | 33.49963 | 291 |
|  | 16.08.2009 | 07:44 | 72.33237 | 33.49962 | 281 |
|  | 16.08.2009 | 10:17 | 71.99985 | 33.50162 | 265 |
|  | 16.08.2009 | 14:47 | 71.79822 | 33.49652 | 328 |
|  | 16.08.2009 | 17:13 | 71.49918 | 33.49915 | 282 |
|  | 16.08.2009 | 20:34 | 71.29985 | 33.49900 | 251 |
|  | 16.08.2009 | 22:57 | 70.99902 | 33.49943 | 223 |
|  | 17.08.2009 | 03:20 | 70.49802 | 33.50437 | 252 |
|  | 17.08.2009 | 07:11 | 69.99843 | 33.50648 | 148 |
|  | 19.08.2009 | 08:15 | 69.50000 | 33.50160 | 268 |
|  | 19.08.2010 | 02:48 | 69.49833 | 33.50500 | 247 |
|  | 19.08.2010 | 07:32 | 69.99000 | 33.50500 | 150 |
|  | 19.08.2010 | 11:45 | 70.49167 | 33.50167 | 258 |
|  | 19.08.2010 | 16:30 | 71.00000 | 33.50000 | 224 |
|  | 19.08.2010 | 19:15 | 71.29982 | 33.50445 | 251 |
|  | 19.08.2010 | 21:00 | 71.49873 | 33.50072 | 281 |
|  | 19.08.2010 | 23:55 | 71.79628 | 33.50442 | 330 |
|  | 20.08.2010 | 01:58 | 71.99992 | 33.49983 | 266 |
|  | 20.08.2010 | 05:00 | 72.33187 | 33.50082 | 292 |
|  | 20.08.2010 | 06:40 | 72.50078 | 33.50317 | 291 |
|  | 20.08.2010 | 11:10 | 72.99992 | 33.50298 | 217 |
|  | 20.08.2010 | 15:40 | 73.49868 | 33.49998 | 286 |
|  | 20.08.2010 | 21:35 | 74.00412 | 33.48878 | 326 |
|  | 21.08.2010 | 06:10 | 74.50035 | 33.50015 | 267 |
|  | 21.08.2010 | 11:00 | 74.99978 | 33.49688 | 147 |
|  | 21.08.2010 | 18:53 | 75.49962 | 33.49948 | 230 |
|  | 22.08.2010 | 00:29 | 76.00087 | 33.49793 | 310 |
|  | 22.08.2010 | 06:11 | 76.50055 | 33.49297 | 215 |
|  | 22.08.2010 | 12:04 | 76.99913 | 33.49633 | 161 |
|  | 22.08.2010 | 22:30 | 76.50215 | 35.59483 | 255 |
|  | 23.08.2010 | 09:30 | 75.79042 | 39.83062 | 315 |
|  | 23.08.2010 | 16:37 | 75.33503 | 42.21375 | 225 |
|  | 23.08.2010 | 21:00 | 75.00038 | 43.81287 | 324 |
|  | 24.08.2010 | 02:46 | 74.54297 | 45.84472 | 312 |
|  | 24.08.2010 | 09:20 | 73.99992 | 47.97225 | 265 |
|  | 24.08.2010 | 16:43 | 73.34157 | 50.24550 | 268 |
|  | 24.08.2010 | 19:00 | 73.36295 | 51.06742 | 192 |
|  | 26.08.2010 | 14:19 | 72.31508 | 50.74722 | 131 |
|  | 26.08.2010 | 19:14 | 72.60022 | 50.68355 | 165 |
|  | 26.08.2010 | 22:00 | 72.43362 | 49.73112 | 109 |
|  | 27.08.2010 | 03:26 | 72.06587 | 48.25795 | 208 |
|  | 27.08.2010 | 06:07 | 71.89893 | 47.86610 | 65 |
|  | 27.08.2010 | 09:55 | 71.59940 | 46.94552 | 55 |
|  | 27.08.2010 | 14:05 | 71.33282 | 45.56638 | 73 |
|  | 27.08.2010 | 16:51 | 71.14763 | 45.04672 | 265 |
|  | 27.08.2010 | 10:00 | 70.99905 | 44.32262 | 144 |
|  | 27.08.2010 | 21:55 | 70.88427 | 43.85408 | 117 |
|  | 28.08.2010 | 01:26 | 70.63265 | 42.95395 | 79 |
|  | 28.08.2010 | 03:22 | 70.50023 | 42.35268 | 63 |
|  | 28.08.2010 | 06:40 | 70.26805 | 41.75172 | 97 |
|  | 28.08.2010 | 08:15 | 70.18373 | 41.33692 | 127 |
|  | 28.08.2010 | 11:55 | 70.04858 | 40.73930 | 108 |
|  | 28.08.2010 | 14:58 | 69.83482 | 39.87965 | 228 |
|  | 28.08.2010 | 20:00 | 69.50080 | 38.55747 | 117 |
|  | 28.08.2010 | 22:55 | 69.26657 | 38.00402 | 235 |
|  | 23.09.2011 | 05:15 | 69.50267 | 33.49842 | 268 |
|  | 23.09.2011 | 13:40 | 69.99733 | 33.48600 | 158 |
|  | 24.09.2011 | 11:27 | 73.99875 | 33.48950 | 215 |
|  | 24.09.2011 | 15:02 | 73.50000 | 33.50002 | 286 |
|  | 24.09.2011 | 21:45 | 74.00092 | 33.48770 | 326 |
|  | 25.09.2011 | 01:38 | 74.49850 | 33.49783 | 268 |
|  | 25.09.2011 | 08:31 | 75.00143 | 33.49677 | 147 |
|  | 25.09.2011 | 13:14 | 75.49995 | 33.49895 | 230 |
|  | 25.09.2011 | 18:30 | 76.00822 | 33.50783 | 305 |
|  | 26.09.2011 | 00:42 | 76.49970 | 33.50297 | 215 |
|  | 26.09.2011 | 05:46 | 77.00283 | 33.53373 | 164 |
|  | 26.09.2011 | 13:11 | 77.49998 | 33.48962 | 150 |
|  | 26.09.2011 | 16:48 | 78.00267 | 33.52135 | 190 |
|  | 28.09.2011 | 15:55 | 72.49540 | 33.50175 | 290 |
|  | 28.09.2011 | 19:50 | 72.34357 | 33.52645 | 286 |
|  | 28.09.2011 | 23:02 | 72.00140 | 33.50000 | 268 |
|  | 29.09.2011 | 01:26 | 71.80108 | 33.50182 | 325 |
|  | 29.09.2011 | 08:08 | 71.49987 | 33.50255 | 285 |
|  | 29.09.2011 | 11:41 | 71.29715 | 33.50515 | 253 |
|  | 29.09.2011 | 14:10 | 71.00058 | 33.49435 | 225 |
|  | 29.09.2011 | 19:52 | 70.49910 | 33.50967 | 253 |
|  | 10.11.2012 | 10:38 | 70.99667 | 33.49333 | 224 |
|  | 10.11.2012 | 14:10 | 71.30000 | 33.49833 | 253 |
|  | 10.11.2012 | 16:22 | 71.50000 | 33.50833 | 282 |
|  | 10.11.2012 | 21:10 | 71.80333 | 33.50667 | 321 |
|  | 10.11.2012 | 22:55 | 71.99667 | 33.49833 | 266 |
|  | 11.11.2012 | 03:10 | 72.33667 | 33.51500 | 285 |
|  | 11.11.2012 | 04:40 | 72.49833 | 33.50167 | 291 |
|  | 11.11.2012 | 11:00 | 72.99833 | 33.50833 | 218 |
|  | 11.11.2012 | 16:15 | 73.48667 | 33.49667 | 282 |
|  | 11.11.2012 | 23:24 | 73.99833 | 33.49667 | 328 |
|  | 12.11.2012 | 08:17 | 74.49333 | 33.47667 | 265 |
|  | 13.11.2012 | 09:14 | 74.99667 | 33.51833 | 150 |
|  | 13.11.2012 | 16:00 | 75.50000 | 33.49500 | 226 |
|  | 13.11.2012 | 22:32 | 75.99500 | 33.50500 | 315 |
|  | 14.11.2012 | 15:08 | 77.00000 | 33.50000 | 160 |
|  | 14.11.2012 | 22:32 | 77.50167 | 33.51167 | 155 |
|  | 15.11.2012 | 04:35 | 78.00333 | 33.50333 | 190 |
|  | 15.11.2012 | 13:00 | 78.49500 | 33.50000 | 243 |
|  | 15.11.2012 | 20:58 | 79.00500 | 33.52000 | 236 |
|  | 16.11.2012 | 20:07 | 76.50000 | 33.56333 | 245 |
|  | 17.11.2012 | 02:10 | 76.00833 | 33.50000 | 310 |
|  | 19.11.2012 | 00:27 | 70.51000 | 33.52333 | 255 |
|  | 19.11.2012 | 08:04 | 70.00167 | 33.52000 | 155 |
|  | 19.11.2012 | 19:25 | 69.49833 | 33.55333 | 240 |
|  | 04.06.2013 | 22:42 | 76.15750 | 38.00000 | 256 |
|  | 05.06.2013 | 22:42 | 76.43111 | 39.23528 | 215 |
|  | 07.06.2013 | 03:20 | 77.07083 | 38.05639 | 190 |
|  | 07.06.2013 | 12:25 | 76.89111 | 39.87889 | 204 |
|  | 08.06.2013 | 04:15 | 76.86056 | 41.71028 | 210 |
|  | 08.06.2013 | 12:15 | 76.70861 | 42.88778 | 238 |
|  | 09.06.2013 | 07:45 | 76.37083 | 40.54278 | 225 |
|  | 09.06.2013 | 22:20 | 76.05222 | 41.06611 | 300 |
|  | 19.06.2013 | 23:13 | 77.58389 | 38.53556 | 245 |
|  | 20.06.2013 | 05:47 | 77.82500 | 38.41000 | 220 |
|  | 20.06.2013 | 11:55 | 78.10444 | 38.69361 | 205 |
|  | 20.06.2013 | 22:19 | 78.59000 | 38.10000 | 205 |
|  | 22.06.2013 | 02:10 | 77.77361 | 41.15222 | 220 |
|  | 22.06.2013 | 10:09 | 77.54333 | 40.32861 | 215 |
|  | 22.06.2013 | 23:00 | 77.26556 | 42.14694 | 245 |
|  | 01.06.2013 | 01:08 | 73.96111 | 37.00000 | 238 |
|  | 02.06.2013 | 15:20 | 74.86056 | 36.98861 | 205 |
|  | 03.06.2013 | 06:23 | 75.44139 | 37.40333 | 160 |
|  | 04.06.2013 | 22:42 | 76.15750 | 38.00000 | 256 |
|  | 09.06.2013 | 22:20 | 76.05222 | 41.06611 | 300 |
|  | 10.06.2013 | 12:55 | 75.87417 | 39.24833 | 235 |
|  | 11.06.2013 | 02:05 | 75.49806 | 40.08750 | 235 |
|  | 12.06.2013 | 02:50 | 74.97056 | 39.34083 | 175 |
|  | 12.06.2013 | 15:30 | 74.46306 | 38.87278 | 195 |
|  | 13.06.2013 | 10:15 | 74.20667 | 38.27083 | 205 |
|  | 13.06.2013 | 21:58 | 73.91667 | 38.63444 | 215 |
|  | 31.05.2013 | 12:45 | 73.20139 | 36.17833 | 223 |
|  | 01.06.2013 | 09:45 | 73.68639 | 37.00000 | 237 |
|  | 01.06.2013 | 16:30 | 73.96111 | 37.00000 | 238 |
|  | 13.06.2013 | 21:58 | 73.91667 | 38.63444 | 215 |
|  | 14.06.2013 | 17:45 | 73.40861 | 38.57972 | 250 |
|  | 15.06.2013 | 06:27 | 72.86639 | 39.57306 | 325 |
|  | 15.06.2013 | 15:05 | 72.36528 | 40.00000 | 355 |
|  | 16.06.2013 | 05:03 | 72.89861 | 38.04194 | 355 |
|  | 26.06.2013 | 01:33 | 72.37889 | 38.30861 | 265 |
|  | 26.06.2013 | 18:06 | 72.39278 | 36.99361 | 265 |
|  | 27.06.2013 | 23:37 | 72.41222 | 35.67306 | 245 |
|  | 28.06.2013 | 14:15 | 72.16333 | 34.73333 | 250 |
|  | 29.06.2013 | 17:11 | 71.66833 | 34.01167 | 250 |
|  | 30.06.2013 | 08:25 | 70.93500 | 33.02167 | 250 |
|  | 08.07.2013 | 10:39 | 71.86611 | 37.55417 | 318 |
|  | 09.07.2013 | 20:52 | 71.87417 | 36.15500 | 270 |
|  | 10.07.2013 | 10:19 | 71.35861 | 35.84194 | 220 |
|  | 10.07.2013 | 20:54 | 71.55722 | 34.83611 | 220 |
|  | 13.07.2013 | 15:20 | 70.86639 | 34.04000 | 194 |
|  | 14.07.2013 | 12:05 | 70.33500 | 32.73333 | 275 |
|  | 10.11.2013 | 10:08 | 70.02033 | 33.53333 | 154 |
|  | 10.11.2013 | 15:00 | 70.25467 | 33.50200 | 247 |
|  | 10.11.2013 | 16:55 | 70.49833 | 33.47833 | 250 |
|  | 10.11.2013 | 21:00 | 70.76000 | 33.51117 | 214 |
|  | 10.11.2013 | 23:04 | 70.99833 | 33.50000 | 220 |
|  | 11.11.2013 | 01:50 | 71.26633 | 33.49817 | 250 |
|  | 11.11.2013 | 03:40 | 71.49833 | 33.51000 | 278 |
|  | 11.11.2013 | 08:15 | 71.75150 | 33.53967 | 325 |
|  | 11.11.2013 | 10:30 | 72.00000 | 33.50000 | 265 |
|  | 11.11.2013 | 12:50 | 72.26050 | 33.50117 | 270 |
|  | 11.11.2013 | 14:45 | 72.49833 | 33.48500 | 287 |
|  | 11.11.2013 | 19:00 | 72.75250 | 33.49800 | 260 |
|  | 11.11.2013 | 20:55 | 72.99833 | 33.50167 | 315 |
|  | 11.11.2013 | 23:20 | 73.25117 | 33.50850 | 192 |
|  | 12.11.2013 | 10:30 | 74.00000 | 33.50167 | 326 |
|  | 12.11.2013 | 15:17 | 74.26317 | 33.48000 | 300 |
|  | 12.11.2013 | 16:20 | 74.50000 | 33.49667 | 318 |
|  | 12.11.2013 | 17:19 | 74.75317 | 33.49717 | 225 |
|  | 13.11.2013 | 00:20 | 74.99833 | 33.52333 | 142 |
|  | 13.11.2013 | 19:21 | 75.26317 | 33.50617 | 190 |
|  | 13.11.2013 | 20:30 | 75.49167 | 33.50500 | 220 |
|  | 13.11.2013 | 21:23 | 75.76163 | 33.47817 | 250 |
|  | 13.11.2013 | 12:00 | 76.00000 | 33.49333 | 306 |
|  | 14.11.2013 | 23:25 | 76.73027 | 29.51383 | 260 |
|  | 21.11.2013 | 14:30 | 75.92000 | 17.71667 | 247 |
|  | 21.11.2013 | 20:00 | 75.72892 | 18.66817 | 91 |
|  | 22.11.2013 | 00:00 | 75.32398 | 19.92500 | 38 |
|  | 22.11.2013 | 04:00 | 75.01202 | 20.90033 | 53 |
|  | 22.11.2013 | 08:00 | 74.76523 | 21.98700 | 158 |
|  | 22.11.2013 | 14:00 | 74.44648 | 22.85217 | 119 |
|  | 23.11.2013 | 00:00 | 74.03567 | 23.39717 | 450 |
|  | 23.11.2013 | 04:00 | 73.79050 | 24.81433 | 468 |
|  | 23.11.2013 | 08:00 | 73.53490 | 26.18050 | 458 |
|  | 23.11.2013 | 12:00 | 73.26200 | 27.32850 | 380 |
|  | 23.11.2013 | 16:00 | 72.86945 | 27.84283 | 300 |
|  | 23.11.2013 | 20:00 | 72.44543 | 28.60200 | 300 |
|  | 24.11.2013 | 00:00 | 72.00567 | 29.34300 | 295 |
|  | 24.11.2013 | 04:00 | 71.55423 | 30.07467 | 320 |
|  | 24.11.2013 | 08:00 | 71.11055 | 30.76767 | 300 |
|  | 24.11.2013 | 12:00 | 70.73508 | 31.65300 | 270 |
|  | 24.11.2013 | 16:00 | 70.39833 | 32.35300 | 196 |
|  | 24.11.2013 | 20:00 | 69.99027 | 32.99883 | 175 |
|  | 25.11.2013 | 00:30 | 69.49713 | 33.52000 | 280 |
|  | 04.11.2015 | 09:30 | 69.49667 | 33.50167 | 271 |
|  | 06.11.2015 | 21:00 | 70.49833 | 33.53000 | 248 |
|  | 07.11.2015 | 01:30 | 70.99833 | 33.50500 | 221 |
|  | 07.11.2015 | 08:10 | 71.30333 | 33.52333 | 250 |
|  | 07.11.2015 | 10:20 | 71.49333 | 33.51667 | 277 |
|  | 07.11.2015 | 14:10 | 71.80167 | 33.49833 | 318 |
|  | 07.11.2015 | 16:50 | 72.00000 | 33.50000 | 264 |
|  | 13.11.2015 | 09:10 | 69.99500 | 33.51500 | 146 |
|  | 17.11.2015 | 09:10 | 77.50167 | 26.98500 | 179 |
|  | 17.11.2015 | 18:50 | 77.00167 | 23.84167 | 76 |
|  | 18.11.2015 | 00:20 | 76.61667 | 22.83667 | 143 |
|  | 18.11.2015 | 08:10 | 76.36167 | 21.50833 | 223 |
|  | 18.11.2015 | 13:20 | 76.16535 | 19.95733 | 192 |
|  | 20.11.2015 | 16:20 | 76.49333 | 33.49167 | 211 |
|  | 21.11.2015 | 00:20 | 77.00147 | 33.52633 | 158 |
|  | 21.11.2015 | 08:30 | 77.50000 | 33.50333 | 148 |
|  | 21.11.2015 | 14:50 | 77.99838 | 33.48950 | 187 |
|  | 21.11.2015 | 21:20 | 78.49833 | 33.50667 | 241 |
|  | 22.11.2015 | 04:40 | 79.00833 | 33.54167 | 234 |
|  | 22.11.2015 | 13:10 | 79.49550 | 33.50500 | 293 |
|  | 22.11.2015 | 20:30 | 79.90167 | 33.54500 | 220 |
|  | 25.11.2015 | 01:30 | 76.08700 | 18.66733 | 197 |
|  | 25.11.2015 | 08:20 | 76.18500 | 16.90167 | 275 |
|  | 25.11.2015 | 21:30 | 77.65000 | 11.72833 | 147 |
|  | 09.12.2015 | 02:50 | 76.01117 | 33.47950 | 306 |
|  | 09.12.2015 | 08:00 | 75.49500 | 33.52333 | 221 |
|  | 09.12.2015 | 12:10 | 75.00133 | 33.49933 | 142 |
|  | 09.12.2015 | 19:20 | 74.50167 | 33.52333 | 265 |
|  | 10.12.2015 | 01:20 | 74.00167 | 33.49233 | 319 |
|  | 10.12.2015 | 07:10 | 73.50000 | 33.52500 | 283 |
|  | 10.12.2015 | 12:30 | 72.99600 | 33.50517 | 215 |
|  | 10.12.2015 | 20:10 | 72.50503 | 33.46750 | 286 |
|  | 10.12.2015 | 22:20 | 72.33480 | 33.48967 | 279 |
|  | 11.12.2015 | 01:50 | 71.99700 | 33.50100 | 261 |
|  | 11.12.2015 | 03:40 | 71.79883 | 33.50383 | 325 |
|  | 11.12.2015 | 06:00 | 71.50000 | 33.49667 | 280 |
|  | 11.12.2015 | 07:50 | 71.30000 | 33.51333 | 250 |
|  | 11.12.2015 | 10:20 | 71.00093 | 33.49183 | 222 |
|  | 11.12.2015 | 14:20 | 70.49633 | 33.48900 | 248 |
|  | 11.12.2015 | 18:00 | 70.00000 | 33.49667 | 152 |
|  | 12.12.2015 | 01:10 | 69.50083 | 33.50433 | 265 |
|  | 06.04.2016 | 04:45 | 69.50083 | 33.50028 | 267 |
|  | 06.04.2016 | 12:40 | 70.00056 | 33.50083 | 145 |
|  | 06.04.2016 | 18:20 | 70.50028 | 33.50111 | 250 |
|  | 07.04.2016 | 01:07 | 71.00000 | 33.50083 | 217 |
|  | 07.04.2016 | 07:05 | 71.28556 | 33.51750 | 250 |
|  | 07.04.2016 | 10:55 | 71.48583 | 33.48583 | 278 |
|  | 07.04.2016 | 16:00 | 71.80056 | 33.55250 | 312 |
|  | 07.04.2016 | 18:00 | 72.00000 | 33.51667 | 267 |
|  | 07.04.2016 | 18:05 | 72.33472 | 33.51667 | 282 |
|  | 09.04.2016 | 19:30 | 72.48556 | 33.51806 | 288 |
|  | 10.04.2016 | 03:02 | 73.00083 | 33.48472 | 216 |
|  | 10.04.2016 | 10:15 | 73.50028 | 33.50000 | 284 |
|  | 10.04.2016 | 17:00 | 74.00028 | 33.50056 | 321 |
|  | 11.04.2016 | 02:15 | 74.50000 | 33.48556 | 261 |
|  | 11.04.2016 | 17:20 | 74.98500 | 33.50056 | 148 |
|  | 12.04.2016 | 22:00 | 76.43417 | 33.70111 | 254 |
|  | 13.04.2016 | 09:27 | 76.43556 | 34.55028 | 247 |
|  | 13.04.2016 | 13:42 | 76.41917 | 34.55083 | 262 |
|  | 13.04.2016 | 14:34 | 76.40083 | 34.56778 | 283 |
|  | 13.04.2016 | 18:15 | 76.35028 | 34.55222 | 275 |
|  | 13.04.2016 | 23:14 | 76.00111 | 34.56750 | 257 |
|  | 14.04.2016 | 12:16 | 76.18556 | 38.93500 | 252 |
|  | 14.04.2016 | 16:15 | 76.16861 | 39.00083 | 252 |
|  | 14.04.2016 | 17:30 | 76.15111 | 39.00000 | 244 |
|  | 14.04.2016 | 20:03 | 76.10139 | 39.00111 | 256 |
|  | 14.04.2016 | 23:28 | 75.75194 | 39.00083 | 245 |
|  | 16.04.2016 | 06:00 | 78.20222 | 43.91889 | 281 |
|  | 16.04.2016 | 14:20 | 78.13500 | 43.88528 | 296 |
|  | 16.04.2016 | 15:01 | 78.11722 | 43.90250 | 297 |
|  | 16.04.2016 | 16:15 | 78.08333 | 43.86917 | 311 |
|  | 16.04.2016 | 22:03 | 77.71750 | 43.98417 | 349 |
|  | 17.04.2016 | 11:00 | 78.15028 | 49.05194 | 300 |
|  | 19.04.2016 | 16:00 | 78.68667 | 49.06167 | 276 |
|  | 19.04.2016 | 21:22 | 78.67333 | 49.05500 | 284 |
|  | 19.04.2016 | 22:10 | 78.64333 | 49.06167 | 269 |
|  | 20.04.2016 | 00:25 | 78.42833 | 49.02833 | 250 |
|  | 20.04.2016 | 02:45 | 78.24500 | 49.06667 | 234 |
|  | 20.04.2016 | 11:00 | 78.52667 | 54.06167 | 300 |
|  | 20.04.2016 | 16:30 | 78.51500 | 54.08833 | 298 |
|  | 20.04.2016 | 17:40 | 78.48333 | 54.06833 | 259 |
|  | 20.04.2016 | 19:15 | 78.43833 | 54.07333 | 282 |
|  | 20.04.2016 | 22:35 | 78.25333 | 54.03833 | 260 |
|  | 21.04.2016 | 02:45 | 78.07500 | 53.99833 | 257 |
|  | 21.04.2016 | 16:00 | 78.95167 | 58.98167 | 260 |
|  | 24.04.2016 | 06:20 | 78.99500 | 58.98000 | 249 |
|  | 24.04.2016 | 08:50 | 78.99000 | 58.95000 | 238 |
|  | 24.04.2016 | 09:30 | 78.95833 | 58.94167 | 238 |
|  | 24.04.2016 | 10:15 | 78.91333 | 58.95000 | 271 |
|  | 24.04.2016 | 12:20 | 78.73000 | 58.94000 | 243 |
|  | 24.04.2016 | 17:45 | 78.55333 | 58.98000 | 174 |
|  | 25.04.2016 | 21:50 | 79.25500 | 64.18000 | 300 |
|  | 26.04.2016 | 02:40 | 79.24167 | 64.17500 | 312 |
|  | 26.04.2016 | 03:26 | 79.20667 | 64.00000 | 302 |
|  | 26.04.2016 | 04:40 | 79.16167 | 64.17667 | 313 |
|  | 26.04.2016 | 06:45 | 78.98500 | 64.13667 | 356 |
|  | 26.04.2016 | 13:32 | 78.81000 | 64.19667 | 365 |
|  | 07.05.2016 | 18:37 | 69.84500 | 39.85500 | 203 |
|  | 08.05.2016 | 00:45 | 70.18333 | 41.31500 | 198 |
|  | 08.05.2016 | 02:17 | 70.26500 | 41.00000 | 92 |
|  | 08.05.2016 | 04:28 | 70.49833 | 42.35167 | 59 |
|  | 08.05.2016 | 06:21 | 70.63167 | 42.95500 | 73 |
|  | 08.05.2016 | 09:05 | 70.88333 | 43.85500 | 116 |
|  | 08.05.2016 | 10:45 | 71.00000 | 44.00000 | 137 |
|  | 08.05.2016 | 16:00 | 71.17667 | 45.17000 | 245 |
|  | 08.05.2016 | 21:30 | 71.32333 | 45.57000 | 68 |
|  | 09.05.2016 | 02:15 | 71.59833 | 46.00000 | 50 |
|  | 09.05.2016 | 05:57 | 71.89833 | 47.84667 | 59 |
|  | 10.05.2016 | 04:35 | 71.90000 | 47.90500 | 61 |
|  | 10.05.2016 | 07:24 | 72.16667 | 48.00000 | 128 |
|  | 11.05.2016 | 11:15 | 72.42667 | 49.74667 | 97 |
|  | 11.05.2016 | 14:20 | 72.60000 | 50.68167 | 161 |
|  | 11.05.2016 | 17:35 | 72.67167 | 51.55667 | 75 |
|  | 11.05.2016 | 19:20 | 72.85000 | 51.72000 | 85 |
|  | 10.05.2016 | 08:00 | 70.75167 | 52.00000 | 156 |
|  | 10.05.2016 | 13:35 | 71.33000 | 51.00500 | 130 |
|  | 10.05.2016 | 18:10 | 71.67500 | 50.65333 | 114 |
|  | 10.05.2016 | 21:22 | 71.99667 | 50.64667 | 122 |
|  | 12.05.2016 | 01:58 | 73.73000 | 52.83500 | 89,5 |
|  | 12.05.2016 | 05:00 | 74.00000 | 53.30167 | 140 |
|  | 12.05.2016 | 09:50 | 74.58333 | 54.69500 | 145 |
|  | 12.05.2016 | 12:25 | 74.91333 | 54.90333 | 154 |
|  | 12.05.2016 | 16:25 | 75.36000 | 55.34167 | 193 |
|  | 12.05.2016 | 19:12 | 75.50667 | 56.12167 | 168 |
|  | 13.05.2016 | 00:15 | 76.00000 | 57.92167 | 93 |
|  | 13.05.2016 | 04:00 | 76.33667 | 59.17667 | 173 |
|  | 13.05.2016 | 07:25 | 76.53000 | 61.01167 | 85 |
|  | 13.05.2016 | 15:34 | 76.88167 | 65.26833 | 224 |
|  | 13.05.2016 | 20:13 | 77.28333 | 67.50167 | 229 |
|  | 13.05.2016 | 23:05 | 77.43333 | 67.21167 | 295 |
|  | 14.05.2016 | 02:13 | 77.83333 | 67.04833 | 365 |
|  | 14.05.2016 | 08:00 | 78.19667 | 64.93667 | 389 |
|  | 16.05.2016 | 23:43 | 78.00000 | 33.48000 | 185 |
|  | 17.05.2016 | 05:48 | 77.49667 | 33.51333 | 155 |
|  | 17.05.2016 | 11:27 | 77.00000 | 33.50667 | 159 |
|  | 17.05.2016 | 16:28 | 76.49833 | 33.51333 | 208 |
|  | 18.05.2016 | 13:55 | 75.49667 | 33.50333 | 222 |
|  | 18.05.2016 | 20:35 | 74.99833 | 33.49500 | 143 |
|  | 13.07.2017 | 11:20 | 70.03667 | 33.50500 | 283 |
|  | 13.07.2017 | 14:35 | 70.49333 | 33.49667 | 218 |
|  | 13.07.2017 | 18:38 | 71.00500 | 33.49667 | 284 |
|  | 13.07.2017 | 21:45 | 71.50000 | 33.51333 | 321 |
|  | 14.07.2017 | 02:30 | 72.00000 | 33.50500 | 305 |
|  | 14.07.2017 | 07:20 | 72.50000 | 33.50000 | 262 |
|  | 14.07.2017 | 12:40 | 73.00500 | 33.51000 | 227 |
|  | 14.07.2017 | 18:20 | 73.50000 | 33.49667 | 143 |
|  | 14.07.2017 | 23:00 | 74.00333 | 33.50500 | 184 |
|  | 15.07.2017 | 02:55 | 74.24833 | 33.50167 | 225 |
|  | 15.07.2017 | 05:50 | 74.50000 | 33.50167 | 230 |
|  | 15.07.2017 | 08:00 | 74.75000 | 33.50000 | 307 |
|  | 15.07.2017 | 10:05 | 75.00167 | 33.50667 | 308 |
|  | 15.07.2017 | 12:50 | 75.24880 | 33.49933 | 208 |
|  | 15.07.2017 | 14:55 | 75.50043 | 33.50033 | 110 |
|  | 15.07.2017 | 17:40 | 75.75115 | 33.50023 | 160 |
|  | 15.07.2017 | 19:40 | 76.00267 | 33.49667 | 144 |
|  | 15.07.2017 | 23:00 | 76.25667 | 33.50388 | 155 |
|  | 16.07.2017 | 00:55 | 76.50140 | 33.50107 | 178 |
|  | 16.07.2017 | 04:25 | 76.75178 | 33.49073 | 185 |
|  | 16.07.2017 | 06:15 | 77.00278 | 33.50430 | 169 |
|  | 16.07.2017 | 10:07 | 77.25303 | 33.50402 | 241 |
|  | 16.07.2017 | 12:15 | 77.50110 | 33.49815 | 289 |
|  | 16.07.2017 | 15:05 | 77.75162 | 33.51117 | 230 |
|  | 16.07.2017 | 17:10 | 78.00228 | 33.52012 | 251 |
|  | 16.07.2017 | 20:32 | 78.25078 | 33.50805 | 240 |
|  | 16.07.2017 | 22:45 | 78.50195 | 33.50130 | 178 |
|  | 17.07.2017 | 02:40 | 78.73957 | 33.51415 | 79 |
|  | 17.07.2017 | 14:45 | 78.35623 | 31.67882 | 80 |
|  | 17.07.2017 | 23:30 | 77.97167 | 30.78272 | 181 |
|  | 18.07.2017 | 04:35 | 77.81725 | 30.83977 | 151 |
|  | 18.07.2017 | 11:40 | 77.50212 | 27.98740 | 272 |
|  | 18.07.2017 | 15:20 | 77.22230 | 25.43230 | 235 |
|  | 18.07.2017 | 19:20 | 76.90100 | 23.60585 | 271 |
|  | 18.07.2017 | 23:10 | 76.60537 | 21.91978 | 149 |
|  | 19.07.2017 | 02:15 | 76.51680 | 20.74863 | 97 |
|  | 19.07.2017 | 04:35 | 76.43458 | 19.58053 | 373 |
|  | 19.07.2017 | 08:05 | 76.35133 | 18.40230 | 85 |
|  | 19.07.2017 | 10:19 | 76.26162 | 17.22322 | 73 |
|  | 19.07.2017 | 17:00 | 76.50942 | 15.17438 | 171 |
|  | 10.11.2017 | 17:12 | 69.50200 | 33.50217 | 265 |
|  | 11.11.2017 | 21:32 | 71.54833 | 25.04650 | 298 |
|  | 12.11.2017 | 00:55 | 71.79617 | 24.57183 | 289 |
|  | 12.11.2017 | 03:40 | 72.03217 | 24.03417 | 290 |
|  | 12.11.2017 | 06:05 | 72.25183 | 23.65267 | 283 |
|  | 12.11.2017 | 08:33 | 72.45583 | 23.20217 | 311 |
|  | 12.11.2017 | 11:32 | 72.71683 | 22.57717 | 395 |
|  | 12.11.2017 | 14:20 | 73.01567 | 21.99100 | 436 |
|  | 12.11.2017 | 17:24 | 73.28567 | 21.44217 | 474 |
|  | 12.11.2017 | 20:47 | 73.58400 | 20.76183 | 501 |
|  | 12.11.2017 | 22:31 | 73.73367 | 20.45200 | 484 |
|  | 13.11.2017 | 00:20 | 73.87400 | 20.14700 | 325 |
|  | 13.11.2017 | 03:20 | 74.01633 | 19.82067 | 136 |
|  | 13.11.2017 | 09:09 | 74.41750 | 17.58467 | 142 |
|  | 13.11.2017 | 14:15 | 74.83200 | 18.65233 | 213 |
|  | 13.11.2017 | 19:23 | 75.13500 | 18.16200 | 77 |
|  | 14.11.2017 | 00:50 | 75.45233 | 17.80183 | 140 |
|  | 14.11.2017 | 05:45 | 75.76983 | 17.46817 | 247 |
|  | 14.11.2017 | 10:16 | 76.00433 | 17.15050 | 322 |
|  | 14.11.2017 | 14:35 | 76.19967 | 16.90433 | 280 |
|  | 27.11.2017 | 21:46 | 78.93217 | 35.00000 | 310 |
|  | 29.11.2017 | 16:44 | 78.58083 | 32.74800 | 303 |
|  | 29.11.2017 | 22:54 | 77.99950 | 33.49550 | 188 |
|  | 30.11.2017 | 01:30 | 77.74733 | 33.47767 | 180 |
|  | 30.11.2017 | 04:05 | 77.49967 | 33.48950 | 153 |
|  | 30.11.2017 | 13:10 | 77.24883 | 33.50367 | 150 |
|  | 30.11.2017 | 15:35 | 76.99917 | 33.51133 | 160 |
|  | 30.11.2017 | 17:52 | 76.75000 | 33.49817 | 115 |
|  | 30.11.2017 | 20:14 | 76.50217 | 33.49650 | 213 |
|  | 30.11.2017 | 22:46 | 76.24917 | 33.49933 | 312 |
|  | 01.12.2017 | 01:10 | 76.50033 | 33.49833 | 212 |
|  | 01.12.2017 | 03:55 | 76.74983 | 33.49583 | 119 |
|  | 01.12.2017 | 06:31 | 77.00217 | 33.50350 | 162 |
|  | 03.12.2017 | 09:16 | 77.00000 | 33.50067 | 310 |
|  | 03.12.2017 | 12:10 | 75.75217 | 33.50783 | 234 |
|  | 03.12.2017 | 14:39 | 75.50033 | 33.49950 | 231 |
|  | 03.12.2017 | 16:58 | 75.25000 | 33.50450 | 188 |
|  | 03.12.2017 | 19:14 | 75.00033 | 33.50300 | 145 |
|  | 03.12.2017 | 21:29 | 75.75033 | 33.50000 | 232 |
|  | 03.12.2017 | 23:50 | 74.49917 | 33.49983 | 268 |
|  | 04.12.2017 | 03:00 | 74.25017 | 33.49967 | 317 |
|  | 04.12.2017 | 06:12 | 74.00183 | 33.50167 | 325 |
|  | 04.12.2017 | 10:59 | 73.75017 | 33.50100 | 332 |
|  | 04.12.2017 | 13:10 | 73.49817 | 33.50983 | 286 |
|  | 04.12.2017 | 16:23 | 73.24883 | 33.50367 | 198 |
|  | 04.12.2017 | 18:34 | 73.00033 | 33.51450 | 218 |
|  | 05.12.2017 | 00:25 | 72.75117 | 33.50117 | 264 |
|  | 05.12.2017 | 03:50 | 72.50250 | 33.51383 | 288 |
|  | 05.12.2017 | 08:09 | 72.24900 | 33.54717 | 267 |
|  | 05.12.2017 | 12:20 | 71.99717 | 33.49617 | 265 |
|  | 05.12.2017 | 14:20 | 71.75000 | 33.50400 | 324 |
|  | 06.12.2017 | 01:02 | 71.50033 | 33.49533 | 282 |
|  | 06.12.2017 | 10:15 | 71.25067 | 33.50117 | 253 |
|  | 06.12.2017 | 16:36 | 70.99950 | 33.50217 | 221 |
|  | 06.12.2017 | 22:30 | 70.83317 | 33.50917 | 223 |
|  | 07.12.2017 | 02:00 | 70.50000 | 33.50000 | 253 |
|  | 07.12.2017 | 10:06 | 70.24883 | 33.50033 | 251 |
|  | 07.12.2017 | 18:13 | 70.00017 | 33.50200 | 150 |
|  | 20.04.2018 | 08:39 | 74.85000 | 33.32000 | 172 |
|  | 20.04.2018 | 17:31 | 75.06000 | 30.47000 | 386 |
|  | 21.04.2018 | 03:41 | 75.15000 | 28.58000 | 336 |
|  | 21.04.2018 | 12:36 | 75.17000 | 26.20000 | 207 |
|  | 21.04.2018 | 16:53 | 75.17000 | 24.92000 | 155 |
|  | 21.04.2018 | 20:24 | 75.17000 | 23.00000 | 107 |
|  | 14.05.2018 | 03:10 | 69.49960 | 33.49890 | 260 |
|  | 14.05.2018 | 06:57 | 70.00030 | 33.50300 | 154 |
|  | 14.05.2018 | 09:00 | 70.25010 | 33.49760 | 253 |
|  | 14.05.2018 | 10:55 | 70.50030 | 33.49800 | 253 |
|  | 14.05.2018 | 12:50 | 70.74970 | 33.49900 | 221 |
|  | 14.05.2018 | 14:45 | 70.99960 | 33.50150 | 224 |
|  | 14.05.2018 | 16:40 | 71.24980 | 33.49830 | 254 |
|  | 14.05.2018 | 18:50 | 71.50000 | 33.51520 | 282 |
|  | 14.05.2018 | 21:00 | 71.74890 | 33.49670 | 320 |
|  | 14.05.2018 | 23:10 | 71.99970 | 33.49810 | 265 |
|  | 15.05.2018 | 01:15 | 72.24940 | 33.49800 | 264 |
|  | 15.05.2018 | 03:10 | 72.49980 | 33.50020 | 292 |
|  | 15.05.2018 | 05:08 | 72.75060 | 33.50170 | 264 |
|  | 15.05.2018 | 07:05 | 73.00040 | 33.50010 | 222 |
|  | 15.05.2018 | 09:40 | 73.25270 | 33.47820 | 203 |
|  | 15.05.2018 | 11:45 | 73.50130 | 33.49720 | 289 |
|  | 15.05.2018 | 14:10 | 73.75160 | 33.49820 | 332 |
|  | 15.05.2018 | 16:16 | 74.00160 | 33.50270 | 325 |
|  | 15.05.2018 | 18:13 | 74.24980 | 33.50170 | 315 |
|  | 15.05.2018 | 20:10 | 74.50050 | 33.49910 | 266 |
|  | 15.05.2018 | 22:00 | 74.75110 | 33.49960 | 233 |
|  | 15.05.2018 | 23:45 | 75.00020 | 33.49770 | 147 |
|  | 16.05.2018 | 01:30 | 75.25100 | 33.49730 | 187 |
|  | 16.05.2018 | 03:10 | 75.50160 | 33.49870 | 230 |
|  | 16.05.2018 | 05:17 | 75.75060 | 33.50280 | 232 |
|  | 16.05.2018 | 07:15 | 76.00010 | 33.50280 | 307 |
|  | 16.05.2018 | 09:15 | 76.25040 | 33.50000 | 311 |
|  | 16.05.2018 | 11:20 | 76.49890 | 33.49860 | 217 |
|  | 16.05.2018 | 13:15 | 76.75040 | 33.49640 | 118 |
|  | 16.05.2018 | 14:55 | 77.00190 | 33.49990 | 160 |
|  | 16.05.2018 | 18:32 | 77.50010 | 33.50120 | 153 |
|  | 19.05.2018 | 17:01 | 78.02667 | 33.50000 | 193 |
|  | 21.05.2018 | 21:35 | 77.50892 | 49.66953 | 397 |
|  | 22.05.2018 | 10:35 | 77.33910 | 49.66550 | 358 |
|  | 22.05.2018 | 19:04 | 77.00763 | 49.66787 | 338 |
|  | 23.05.2018 | 00:40 | 76.84222 | 49.66877 | 307 |
|  | 23.05.2018 | 04:38 | 76.67677 | 49.67045 | 290 |
|  | 03.04.2019 | 16:10 | 69.98840 | 33.49265 | 151 |
|  | 03.04.2019 | 20:40 | 70.25578 | 33.50118 | 248 |
|  | 03.04.2019 | 22:40 | 70.50235 | 33.50617 | 250 |
|  | 04.04.2019 | 02:50 | 70.76758 | 33.50030 | 216 |
|  | 04.04.2019 | 05:10 | 71.00423 | 33.50128 | 215 |
|  | 04.04.2019 | 11:37 | 71.24855 | 33.50215 | 249 |
|  | 04.04.2019 | 14:17 | 71.49588 | 33.50338 | 278 |
|  | 04.04.2019 | 20:10 | 71.74765 | 33.50737 | 317 |
|  | 04.04.2019 | 22:54 | 72.00515 | 33.48335 | 266 |
|  | 05.04.2019 | 03:30 | 72.24917 | 33.50098 | 264 |
|  | 05.04.2019 | 06:30 | 72.50815 | 33.50000 | 284 |
|  | 05.04.2019 | 11:10 | 72.75203 | 33.49925 | 258 |
|  | 07.04.2019 | 20:35 | 73.00000 | 33.50000 | 214 |
|  | 08.04.2019 | 18:40 | 73.25397 | 33.50273 | 208 |
|  | 08.04.2019 | 20:50 | 73.50708 | 33.48817 | 287 |
|  | 09.04.2019 | 00:05 | 73.75135 | 33.49002 | 328 |
|  | 09.04.2019 | 02:10 | 74.00140 | 33.49917 | 318 |
|  | 09.04.2019 | 06:40 | 74.25150 | 33.50757 | 318 |
|  | 09.04.2019 | 08:45 | 74.50308 | 33.50035 | 260 |
|  | 09.04.2019 | 10:45 | 74.74812 | 33.49703 | 230 |
|  | 09.04.2019 | 12:50 | 75.00438 | 33.49705 | 144 |
|  | 09.04.2019 | 16:50 | 75.17540 | 33.52533 | 180 |
|  | 11.04.2019 | 09:10 | 75.10243 | 37.98485 | 187 |
|  | 11.04.2019 | 21:30 | 74.93125 | 38.02695 | 180 |
|  | 12.04.2019 | 01:27 | 74.77152 | 37.99855 | 155 |
|  | 12.04.2019 | 04:05 | 74.60568 | 37.99730 | 148 |
|  | 13.04.2019 | 09:05 | 75.74790 | 43.00062 | 285 |
|  | 13.04.2019 | 20:30 | 75.58255 | 43.01313 | 318 |
|  | 13.04.2019 | 22:30 | 75.41483 | 43.00818 | 294 |
|  | 14.04.2019 | 00:45 | 75.25090 | 42.99863 | 280 |
|  | 14.04.2019 | 20:10 | 76.53708 | 49.66583 | 290 |
|  | 15.04.2019 | 05:00 | 76.36522 | 49.67152 | 246 |
|  | 15.04.2019 | 07:15 | 76.19962 | 49.66972 | 245 |
|  | 15.04.2019 | 09:20 | 76.03462 | 49.67928 | 235 |
|  | 17.04.2019 | 03:05 | 71.83518 | 38.00520 | 340 |
|  | 17.04.2019 | 06:50 | 71.49925 | 37.74365 | 295 |
|  | 17.04.2019 | 12:05 | 71.26533 | 37.66485 | 270 |
|  | 17.04.2019 | 14:00 | 71.07873 | 37.54402 | 226 |
|  | 17.04.2019 | 16:05 | 70.83167 | 37.32575 | 178 |
|  | 18.04.2019 | 04:20 | 70.50235 | 37.23708 | 185 |
|  | 18.04.2019 | 11:40 | 70.16142 | 37.00232 | 169 |
|  | 18.04.2019 | 14:20 | 69.83695 | 36.79148 | 126 |
|  | 18.04.2019 | 20:05 | 69.49638 | 36.67922 | 204 |
|  | 18.04.2019 | 21:00 | 69.42698 | 36.43385 | 200 |
|  | 23.09.2019 | n/a | 68.62467 | 48.23083 | 52 |
|  | 24.09.2019 | n/a | 68.87750 | 46.60117 | 63 |
|  | 24.09.2019 | n/a | 69.07593 | 44.55790 | 61 |
|  | 25.09.2019 | n/a | 69.24945 | 40.25865 | 148 |
|  | 25.09.2019 | n/a | 69.35565 | 36.99320 | 199 |
|  | 26.09.2019 | 08:40 | 69.48408 | 33.82462 | 214 |
|  | 19.06.2019 | 01:15 | 70.00000 | 33.50000 | 147 |
|  | 19.06.2019 | 05:20 | 70.25000 | 33.50000 | 248 |
|  | 19.06.2019 | 07:55 | 70.50000 | 33.50000 | 250 |
|  | 19.06.2019 | 11:21 | 70.75000 | 33.50000 | 218 |
|  | 19.06.2019 | 13:40 | 71.00000 | 33.50000 | 223 |
|  | 19.06.2019 | 16:40 | 71.25000 | 33.50000 | 250 |
|  | 19.06.2019 | 20:10 | 71.50000 | 33.50000 | 279 |
|  | 20.06.2019 | 02:25 | 71.75000 | 33.50000 | 316 |
|  | 20.06.2019 | 06:10 | 72.00000 | 33.50000 | 263 |
|  | 20.06.2019 | 10:30 | 72.25000 | 33.50000 | 262 |
|  | 20.06.2019 | 12:45 | 72.50000 | 33.50000 | 288 |
|  | 20.06.2019 | 16:30 | 72.75000 | 33.50000 | 258 |
|  | 20.06.2019 | 18:50 | 73.00000 | 33.50000 | 215 |
|  | 20.06.2019 | 22:20 | 73.25000 | 33.50000 | 200 |
|  | 21.06.2019 | 00:30 | 73.50000 | 33.50000 | 285 |
|  | 21.06.2019 | 06:15 | 73.75000 | 33.50000 | 329 |
|  | 21.06.2019 | 09:05 | 74.00000 | 33.50000 | 315 |
|  | 21.06.2019 | 13:10 | 74.25000 | 33.50000 | 316 |
|  | 21.06.2019 | 16:10 | 74.50000 | 33.50000 | 262 |
|  | 21.06.2019 | 20:17 | 74.75000 | 33.50000 | 230 |
|  | 21.06.2019 | 23:15 | 75.00000 | 33.50000 | 143 |
|  | 22.06.2019 | 02:20 | 75.25000 | 33.50000 | 183 |
|  | 22.06.2019 | 04:25 | 75.50000 | 33.50000 | 226 |
|  | 22.06.2019 | 06:35 | 75.75000 | 33.50000 | 229 |
|  | 22.06.2019 | 08:41 | 76.00000 | 33.50000 | 308 |
|  | 22.06.2019 | 12:10 | 76.25000 | 33.50000 | 309 |
|  | 20.11.2019 | 05:10 | 77.76812 | 11.71857 | 125 |
|  | 20.11.2019 | 10:03 | 78.10887 | 9.98773 | 211 |
|  | 20.11.2019 | 16:10 | 78.55238 | 8.88860 | 830 |
|  | 20.11.2019 | 20:08 | 79.04772 | 8.68440 | 241 |
|  | 21.11.2019 | 01:50 | 79.54550 | 8.70107 | 332 |
|  | 21.11.2019 | 08:03 | 80.04318 | 8.70107 | 505 |
|  | 29.10.2019 | 05:30 | 79.93533 | 70.93817 | 568 |
|  | 29.10.2019 | 14:40 | 79.39500 | 71.01833 | 520 |
|  | 29.10.2019 | 23:25 | 78.75717 | 72.50183 | 502 |
|  | 30.10.2019 | 06:15 | 78.13667 | 72.99500 | 427 |
|  | 30.10.2019 | 13:30 | 77.48890 | 72.02540 | 307 |
|  | 30.10.2019 | 22:51 | 78.13720 | 69.90600 | 450 |
|  | 31.10.2019 | 08:07 | 78.74540 | 69.76180 | 515 |
|  | 31.10.2019 | 17:00 | 79.39790 | 69.36430 | 542 |
|  | 31.10.2019 | 22:34 | 79.39610 | 65.47960 | 437 |
|  | 01.11.2019 | 05:15 | 79.35970 | 62.66810 | 199 |
|  | 01.11.2019 | 14:10 | 79.10000 | 63.45000 | 277 |
|  | 01.11.2019 | 20:17 | 78.77770 | 64.23580 | 365 |
|  | 02.11.2019 | 04:15 | 78.74720 | 66.79980 | 346 |
|  | 02.11.2019 | 10:50 | 78.50018 | 65.32558 | 375 |
|  | 02.11.2019 | 17:50 | 78.13630 | 66.57793 | 385 |
|  | 03.11.2019 | 01:00 | 77.80000 | 67.60000 | 437 |
|  | 03.11.2019 | 07:00 | 77.49850 | 68.43200 | 505 |
|  | 03.11.2019 | 17:50 | 77.09213 | 65.14070 | 102 |
|  | 04.11.2019 | 00:40 | 77.61750 | 64.28260 | 271 |
|  | 04.11.2019 | 05:10 | 78.13760 | 62.79590 | 378 |
|  | 04.11.2019 | 11:33 | 78.44013 | 62.00035 | 187 |
|  | 04.11.2019 | 14:20 | 78.74710 | 61.12360 | 188 |
|  | 04.11.2019 | 22:30 | 78.13685 | 58.78683 | 323 |
|  | 05.11.2019 | 05:40 | 77.50330 | 60.19710 | 268 |
|  | 06.11.2019 | 04:10 | 76.88742 | 58.35633 | 205 |
|  | 06.11.2019 | 14:00 | 76.88548 | 62.06050 | 166 |
|  | 06.11.2019 | 18:50 | 76.53240 | 60.96120 | 82 |
|  | 07.11.2019 | 00:55 | 76.40162 | 59.86230 | 123 |
|  | 07.11.2019 | 12:25 | 75.60403 | 56.55815 | 155 |
