# Supplementary material for: Findings of new phytoplankton species in the Barents Sea as a consequence of global climate changes
Source: PeerJ. 2023 Jun 13;11:e15472. doi: 10.7717/peerj.15472 (PMC10274593; doi:10.7717/peerj.15472)
Supplement: Supplemental Information 2 [file peerj-11-15472-s002.doc]

**Table A2:**

List of pelagic microalgae species first observed in the Barents Sea

| Date,  DD-MM-YYYY | Latitude, XX.XX° | Longitude, XX.XX ° | Total depth, m | Sampling depth, m | Quantity, cells/l | Total quantity, cells/l | Dominants |
| --- | --- | --- | --- | --- | --- | --- | --- |
| ***Amphidoma caudata* Halldal** | | | | | | | |
| 10-11-2012 | 71.50 | 33.51 | 282 | 25 | 20 | 648 | *Oxytoxum caudatum*, *Coccolithus pelagicus* |
| 10-11-2013 | 70.02 | 33.53 | 154 | 121-69 | * | - | *Protoperidinium pyriforme* f.breve, *Protoperidinium curtipes*, *Halosphaera viridis* |
| 11-11-2013 | 71.50 | 33.51 | 278 | 40-0 | * | - | *Halosphaera viridis*, *Dictyocha speculum*, *Ceratium tripos*, *Ceratium fusus* |
| ***Ceratium strictum* Kofoid** | | | | | | | |
| 07-06-2014 | 72.45 | 23.20 | 301 | 14 | 5 | 1 136 | *Prorocentrum* sp., *Nitzschia seriata* |
| 10-06-2014 | 69.50 | 33.50 | 270 | 3 | 4 | 219 | *Protoperidinium depressum*, *Protoperidinium pellucidum* |
| 24-07-2015 | 69.54 | 33.03 | 78 | 0 | 15 | 10 000 000 | *Pontosphaera huxleyi* |
| 13-11-2015 | 69.97 | 33.45 | 150 | 0 | 2 | 563 | *Pontosphaera huxleyi*, *Oxytoxum caudatum* |
| 13-11-2015 | 69.97 | 33.45 | 150 | 25 | 1 | 1 053 | *Pontosphaera huxleyi*, *Oxytoxum caudatum* |
| 13-11-2015 | 69.97 | 33.45 | 150 | 100 | 2 | 312 | *Pontosphaera huxleyi* |
| 21-11-2015 | 78.50 | 33.51 | 230 | 55 | 2 | 71 | *Protoperidinium depressum*, *Coccolithus pelagicus*, *Prorocentrum* sp. |
| 20-06-2019 | 72.50 | 33.50 | 288 | 39-0 | * | - | *Chaetoceros concavicornis* |
| 21-06-2019 | 74.50 | 33.50 | 262 | 0 | 4 | 144 091 | *Attheya longicornis*, *Chaetoceros concavicornis*, *Chaetoceros diadema*, *Dinobryon balticum* |
| ***Corythodinium diploconus* Taylor** | | | | | | | |
| 10-11-2012 | 71.50 | 33.51 | 282 | 50 | 1 | 265 | *Oxytoxum caudatum* |
| 10-11-2013 | 70.50 | 33.48 | 250 | 65-0 | * | - | *Ceratium tripos*, *Ceratium fusus*, *Ceratium longipes*, *Halosphaera viridis* |
| 11-11-2013 | 71.50 | 33.51 | 278 | 40-0 | * | - | *Halosphaera viridis*, *Dictyocha speculum*, *Ceratium tripos*, *Ceratium fusus* |
| 11-11-2013 | 71.50 | 33.51 | 278 | 240-155 | * | - | *Dinophysis rotundata*, *Dinophysis norvegica*, *Protoperidinium pyriforme* f.breve, *Ceratium arcticum* |
| 11-11-2013 | 72.50 | 33.48 | 287 | 10 | 3 | 621 | *Oxytoxum caudatum*, *Aulacoseira granulata* |
| ***Dinophysis hastata* Stein** | | | | | | | |
| 10-11-2013 | 70.02 | 33.53 | 154 | 121-69 | * | - | *Protoperidinium pyriforme* f.breve, *Protoperidinium curtipes*, *Halosphaera viridis* |
| 10-11-2013 | 70.50 | 33.48 | 250 | 170-100 | * | - | *Protoperidinium pyriforme* f.breve, *Halosphaera viridis*, *Dinophysis rotundata* |
| ***Dinophysis ovata* Claparede et Lachmann** | | | | | | | |
| 10-11-2013 | 70.02 | 33.53 | 154 | 100 | 2 | 66 | *Dicroerisma psilonereiella*, |
| 10-11-2013 | 70.02 | 33.53 | 154 | 121-69 | * | - | *Protoperidinium pyriforme* f.breve, *Protoperidinium curtipes*, *Halosphaera viridis* |
| 10-11-2013 | 70.50 | 33.48 | 250 | 65-0 | * | - | *Ceratium tripos*, *Ceratium fusus*, *Ceratium longipes*, *Halosphaera viridis* |
| 10-11-2013 | 70.50 | 33.48 | 250 | 170-100 | * | - | *Protoperidinium pyriforme* f.breve, *Halosphaera viridis*, *Dinophysis rotundata* |
| 11-11-2013 | 71.50 | 33.51 | 278 | 240-155 | * | - | *Dinophysis rotundata*, *Dinophysis norvegica*, *Protoperidinium pyriforme* f.breve, *Ceratium arcticum* |
| 11-11-2013 | 72.50 | 33.48 | 287 | 50 | 2 | 81 | *Lessardia elongata* aff., *Pronoctiluca pelagica* |
| 11-11-2013 | 72.50 | 33.48 | 287 | 60-0 | * | - | *Halosphaera viridis*, *Ceratium fusus* |
| 12-11-2013 | 74.50 | 33.50 | 257 | 70-0 | * | - | *Ceratium fusus*, *Ceratium lineatum* |
| 07-06-2014 | 71.55 | 25.03 | 289 | 289 | 4 | 220 | *Thalassionema nitzschioides* |
| 13-11-2015 | 69.97 | 33.45 | 150 | 25 | 2 | 1 053 | *Pontosphaera huxleyi*, *Oxytoxum caudatum* |
| 06-04-2016 | 70.00 | 33.51 | 140 | 50-0 | * | - | *Chaetoceros socialis*, *Chaetoceros furcellatus*, *Chaetoceros diadema*, *Thalassiosira gravida-antarctica*, *Thalassiosira hyalina* aff. |
| 06-04-2016 | 70.00 | 33.51 | 140 | 120-50 | * | - | *Thalassiosira hyalina* aff., *Nitzschia grunowii* |
| 13-04-2016 | 76.35 | 34.56 | 280 | 52-0 | * | - | *Phaeocystis pouchetii*, *Chaetoceros socialis*, *Thalassiosira gravida-antarctica*, *Thalassiosira hyalina* aff. |
| 16-04-2016 | 78.21 | 43.93 | 270 | 50-0 | * | - | *Phaeocystis pouchetii*, *Porosira glacialis* |
| ***Gotoius mutsuensis* Matsuoka** | | | | | | | |
| 07-06-2014 | 71.55 | 25.03 | 289 | 0 | 4 | 270 | *Ceratium longipes*, *Protoperidinium depressum*, *Dinophysis rotundata* |
| ***Heterodinium milneri* Kofoid** | | | | | | | |
| 10-11-2013 | 70.02 | 33.53 | 154 | 121-69 | * | - | *Protoperidinium pyriforme* f.breve, *Protoperidinium curtipes*, *Halosphaera viridis* |
| ***Mesoporos perforatus* Lillick** | | | | | | | |
| 11-11-2013 | 71.50 | 33.51 | 278 | 240-155 | * | - | *Dinophysis rotundata*, *Dinophysis norvegica*, *Protoperidinium pyriforme* f.breve, *Ceratium arcticum* |
| 11-11-2013 | 72.50 | 33.48 | 287 | 10 | 13 | 621 | *Oxytoxum caudatum*, *Aulacoseira granulata* |
| 12-11-2013 | 74.00 | 33.50 | 326 | 0 | 12 | 110 | *Oxytoxum caudatum*, *Mesoporos perforatus*, *Coccolithus pelagicus* |
| 12-11-2013 | 74.00 | 33.50 | 326 | 10 | 7 | 110 | *Oxytoxum caudatum*, *Coccolithus pelagicus* |
| 12-11-2013 | 74.00 | 33.50 | 326 | 25 | 4 | 119 | *Coccolithus pelagicus*, *Oxytoxum caudatum* |
| 12-11-2013 | 74.00 | 33.50 | 326 | 50 | 6 | 53 | *Oxytoxum caudatum* |
| 12-11-2013 | 74.00 | 33.50 | 326 | 200 | 5 | 28 | *Lessardia elongata* aff., *Pronoctiluca pelagica* |
| 12-11-2013 | 74.00 | 33.50 | 326 | 320 | 1 | 54 | *Lessardia elongata* aff., *Protoperidinium pyriforme* f.breve, *Pronoctiluca pelagica* |
| 12-11-2013 | 74.50 | 33.50 | 257 | 10 | 19 | 151 | *Oxytoxum caudatum* |
| 12-11-2013 | 74.50 | 33.50 | 257 | 25 | 16.4 | 101 | *Oxytoxum caudatum*, *Mesoporos perforatus*, *Coccolithus pelagicus* |
| 12-11-2013 | 74.50 | 33.50 | 257 | 50 | 42 | 139 | *Oxytoxum caudatum*, *Mesoporos perforatus* |
| 12-11-2013 | 74.50 | 33.50 | 257 | 100 | 8 | 48 | *Mesoporos perforatus*, *Coccolithus pelagicus* |
| 12-11-2013 | 74.50 | 33.50 | 257 | 200 | 4 | 81 | *Ceratium arcticum* |
| 13-11-2013 | 76.02 | 33.48 | 306 | 0 | 45 | 95 | *Mesoporos perforatus* |
| 13-11-2013 | 76.02 | 33.48 | 306 | 25 | 39 | 64 | *Mesoporos perforatus* |
| 13-11-2013 | 76.02 | 33.48 | 306 | 50 | 33 | 74 | *Mesoporos perforatus*, *Coccolithus pelagicus* |
| 13-11-2013 | 76.02 | 33.48 | 306 | 100 | 47 | 81 | *Mesoporos perforatus* |
| 13-11-2013 | 76.02 | 33.48 | 306 | 173 | 1 | 17 | *Lessardia elongata* aff., *Ceratium arcticum* |
| 13-11-2013 | 76.02 | 33.48 | 306 | 212 | 1 | 39 | *Lessardia elongata* aff., *Dinophysis rotundata* |
| ***Oxytoxum caudatum* Schiller** | | | | | | | |
| 23-08-2007 | 74.50 | 35.77 | 260 | 0 | 500 | 147 750 | *Chaetoceros furcellatus* aff., *Leptocylindrus minimus* |
| 23-08-2007 | 74.50 | 35.77 | 260 | 25 | 100 | 100 059 | *Chaetoceros furcellatus* aff. |
| 23-08-2007 | 74.50 | 35.77 | 260 | 35 | 70 | 86 133 | *Chaetoceros furcellatus* aff. |
| 25-08-2007 | 79.50 | 35.00 | 320 | 0 | 17 | 49 102 | *Dinobryon balticum* |
| 25-08-2007 | 79.50 | 35.00 | 320 | 70 | 3 | 124 | *Chaetoceros furcellatus* |
| 25-08-2007 | 79.50 | 35.00 | 320 | 320 | 50 | 6 085 | *Chaetoceros furcellatus* |
| 26-08-2007 | 80.95 | 38.93 | 225 | 0 | 7 | 20 875 | *Dinobryon balticum* |
| 26-08-2007 | 80.95 | 38.93 | 225 | 6 | 10 | 9 063 | *Dinobryon balticum* |
| 27-08-2007 | 80.93 | 40.74 | 570 | 0 | 29 | 17 201 | *Dinobryon balticum* |
| 27-08-2007 | 80.93 | 40.74 | 570 | 5 | 3 | 31 635 | *Dinobryon balticum* |
| 27-08-2007 | 80.93 | 40.74 | 570 | 130 | 10 | 13 109 | *Dinobryon balticum* |
| 27-08-2007 | 80.93 | 40.74 | 570 | 270 | 13 | 4 516 | *Dinobryon balticum* |
| 28-08-2007 | 80.90 | 42.80 | 575 | 10 | 17 | 226 278 | *Dinobryon balticum* |
| 28-08-2007 | 80.90 | 42.80 | 575 | 35 | 2 | 1 256 753 | *Dinobryon balticum* |
| 28-08-2007 | 80.87 | 46.16 | 210 | 0 | 70 | 606 996 | *Dinobryon balticum* |
| 28-08-2007 | 80.87 | 46.16 | 210 | 5 | 300 | 91 490 | *Dinobryon balticum* |
| 30-08-2007 | 80.34 | 52.77 | 12 | 0 | 30 | 331 015 | *Dinobryon balticum*, *Chaetoceros furcellatus*, *Attheya longicornis* |
| 11-10-2010 | 69.21 | 35.27 | 23 | 0 | 200 | 9 070 | *Pentapharsodinium dalei*, *Leptocylindrus minimus*, L. danicus |
| 11-10-2010 | 69.21 | 35.27 | 23 | 23 | 50 | 8 092 | *Skeletonema costatum*, *Leptocylindrus minimus*, *Chaetoceros affinis* willei, Ch. brevis |
| 19-11-2012 | 70.01 | 33.55 | 147 | 5 | 108 | 153 | *Oxytoxum caudatum* |
| 19-11-2012 | 70.01 | 33.55 | 147 | 50 | 62 | 109 | *Oxytoxum caudatum* |
| 19-11-2012 | 70.01 | 33.55 | 147 | 147 | 5 | 111 | *Lessardia elongata* aff., *Pronoctiluca pelagica* |
| 19-11-2012 | 70.51 | 33.52 | 255 | 0 | 40 | 84 | *Oxytoxum caudatum* |
| 19-11-2012 | 70.51 | 33.52 | 255 | 50 | 90 | 168 | *Oxytoxum caudatum*, *Lessardia elongata* aff. |
| 19-11-2012 | 70.51 | 33.52 | 255 | 100 | 2 | 67 | *Lessardia elongata* aff., *Pronoctiluca pelagica* |
| 10-11-2012 | 71.50 | 33.51 | 282 | 0 | 98 | 304 | *Prorocentrum* sp. |
| 10-11-2012 | 71.50 | 33.51 | 282 | 10 | 315 | 1 126 | *Oxytoxum caudatum* |
| 10-11-2012 | 71.50 | 33.51 | 282 | 25 | 157 | 648 | *Oxytoxum caudatum*, *Coccolithus pelagicus* |
| 10-11-2012 | 71.50 | 33.51 | 282 | 50 | 132 | 265 | *Oxytoxum caudatum* |
| 10-11-2012 | 71.50 | 33.51 | 282 | 100 | 39 | 327 | *Lessardia elongata* aff., *Coccolithus pelagicus* |
| 11-11-2012 | 72.50 | 33.50 | 291 | 0 | 29 | 551 | *Coccolithus pelagicus*, *Prorocentrum* sp. |
| 11-11-2012 | 72.50 | 33.50 | 291 | 10 | 16 | 143 | *Oxytoxum caudatum*, *Prorocentrum* sp. |
| 11-11-2012 | 72.50 | 33.50 | 291 | 25 | 10 | 253 | *Prorocentrum minimum*, *Dinobryon balticum* |
| 11-11-2012 | 72.50 | 33.50 | 291 | 50 | 15 | 139 | *Oxytoxum caudatum*, *Lessardia elongata* aff., *Prorocentrum* sp. |
| 11-11-2012 | 72.50 | 33.50 | 291 | 100 | 67 | 491 | *Heterocapsa triquetra*, *Oxytoxum caudatum*, *Prorocentrum* sp. |
| 11-11-2012 | 72.50 | 33.50 | 291 | 280 | 9 | 222 | *Coccolithus pelagicus*, *Lessardia elongata* aff., *Prorocentrum minimum* |
| 16-11-2012 | 76.50 | 33.56 | 245 | 25 | 4 | 123 | *Ceratium fusus* |
| 10-11-2013 | 70.02 | 33.53 | 154 | 0 | 175 | 234 | *Oxytoxum caudatum* |
| 10-11-2013 | 70.02 | 33.53 | 154 | 10 | 184 | 217 | *Oxytoxum caudatum* |
| 10-11-2013 | 70.02 | 33.53 | 154 | 25 | 184 | 221 | *Oxytoxum caudatum* |
| 10-11-2013 | 70.02 | 33.53 | 154 | 50 | 102 | 132 | *Oxytoxum caudatum* |
| 10-11-2013 | 70.02 | 33.53 | 154 | 100 | 5 | 66 | *Dicroerisma psilonereiella* |
| 10-11-2013 | 70.50 | 33.48 | 250 | 0 | 66 | 148 | *Oxytoxum caudatum* |
| 10-11-2013 | 70.50 | 33.48 | 250 | 10 | 24 | 44 | *Oxytoxum caudatum* |
| 10-11-2013 | 70.50 | 33.48 | 250 | 25 | 137 | 202 | *Oxytoxum caudatum* |
| 10-11-2013 | 70.50 | 33.48 | 250 | 50 | 104 | 152 | *Oxytoxum caudatum* |
| 11-11-2013 | 71.50 | 33.51 | 278 | 0 | 27 | 51 | *Oxytoxum caudatum* |
| 11-11-2013 | 71.50 | 33.51 | 278 | 10 | 109 | 145 | *Oxytoxum caudatum* |
| 11-11-2013 | 71.50 | 33.51 | 278 | 25 | 71 | 91 | *Oxytoxum caudatum* |
| 11-11-2013 | 71.50 | 33.51 | 278 | 50 | 69 | 96 | *Oxytoxum caudatum*, *Thalassionema nitzschioides* |
| 11-11-2013 | 71.50 | 33.51 | 278 | 200 | 1 | 14 | *Coccolithus pelagicus* |
| 11-11-2013 | 72.50 | 33.48 | 287 | 10 | 420 | 621 | *Oxytoxum caudatum*, *Aulacoseira granulata* |
| 11-11-2013 | 72.50 | 33.48 | 287 | 25 | 201 | 279 | *Oxytoxum caudatum* |
| 11-11-2013 | 72.50 | 33.48 | 287 | 100 | 3 | 46 | *Corethron hystrix*, *Halosphaera viridis*, *Lessardia elongata* aff. |
| 11-11-2013 | 72.50 | 33.48 | 287 | 200 | 1 | 37 | *Prorocentrum* sp., *Lessardia elongata* aff. |
| 12-11-2013 | 74.00 | 33.50 | 326 | 0 | 49 | 110 | *Oxytoxum caudatum*, *Mesoporos perforatus*, *Coccolithus pelagicus* |
| 12-11-2013 | 74.00 | 33.50 | 326 | 10 | 66 | 110 | *Oxytoxum caudatum*, *Coccolithus pelagicus* |
| 12-11-2013 | 74.00 | 33.50 | 326 | 25 | 42 | 119 | *Coccolithus pelagicus*, *Oxytoxum caudatum* |
| 12-11-2013 | 74.00 | 33.50 | 326 | 50 | 30 | 53 | *Oxytoxum caudatum* |
| 12-11-2013 | 74.50 | 33.50 | 257 | 0 | 17 | 91 | *Aulacoseira granulata*, *Coccolithus pelagicus*, *Oxytoxum caudatum* |
| 12-11-2013 | 74.50 | 33.50 | 257 | 10 | 81 | 151 | *Oxytoxum caudatum* |
| 12-11-2013 | 74.50 | 33.50 | 257 | 25 | 36.5 | 101 | *Oxytoxum caudatum*, *Mesoporos perforatus*, *Coccolithus pelagicus* |
| 12-11-2013 | 74.50 | 33.50 | 257 | 50 | 51 | 139 | *Oxytoxum caudatum*, *Mesoporos perforatus* |
| 12-11-2013 | 74.50 | 33.50 | 257 | 100 | 5 | 48 | *Mesoporos perforatus*, *Coccolithus pelagicus* |
| 13-11-2013 | 76.02 | 33.48 | 306 | 0 | 7 | 95 | *Mesoporos perforatus* |
| 13-11-2013 | 76.02 | 33.48 | 306 | 25 | 3 | 64 | *Mesoporos perforatus* |
| 13-11-2013 | 76.02 | 33.48 | 306 | 50 | 4 | 74 | *Mesoporos perforatus, Coccolithus pelagicus* |
| 13-11-2013 | 76.02 | 33.48 | 306 | 100 | 5 | 81 | *Mesoporos perforatus* |
| 10-06-2014 | 70.00 | 33.50 | 147 | 147 | 1 | 99 | *Aulacoseira granulata* |
| 04-11-2015 | 69.49 | 33.54 | 281 | 0 | 270 | 3 180 | *Pontosphaera huxleyi, Oxytoxum caudatum* |
| 04-11-2015 | 69.49 | 33.54 | 281 | 25 | 290 | 369 | *Oxytoxum caudatum* |
| 04-11-2015 | 69.49 | 33.54 | 281 | 55 | 330 | 427 | *Oxytoxum caudatum* |
| 04-11-2015 | 69.49 | 33.54 | 281 | 120 | 57 | 1 797 | *Pontosphaera huxleyi* |
| 13-11-2015 | 69.97 | 33.45 | 150 | 0 | 140 | 563 | *Pontosphaera huxleyi, Oxytoxum caudatum* |
| 13-11-2015 | 69.97 | 33.45 | 150 | 10 | 320 | 1 222 | *Pontosphaera huxleyi, Oxytoxum caudatum* |
| 13-11-2015 | 69.97 | 33.45 | 150 | 25 | 220 | 1 053 | *Pontosphaera huxleyi, Oxytoxum caudatum* |
| 13-11-2015 | 69.97 | 33.45 | 150 | 50 | 190 | 1 139 | *Pontosphaera huxleyi, Oxytoxum caudatum* |
| 13-11-2015 | 69.97 | 33.45 | 150 | 100 | 32 | 312 | *Pontosphaera huxleyi* |
| 13-11-2015 | 70.50 | 33.50 | 248 | 100 | 130 | 2 831 | *Pontosphaera huxleyi* |
| 07-11-2015 | 72.01 | 33.50 | 265 | 0 | 140 | 574 | *Oxytoxum caudatum, Pontosphaera huxleyi, Prorocentrum* sp. |
| 07-11-2015 | 72.01 | 33.50 | 265 | 25 | 54 | 2 590 | *Pontosphaera huxleyi* |
| 07-11-2015 | 72.01 | 33.50 | 265 | 80 | 16 | 1 201 | *Pontosphaera huxleyi, Coccolithus pelagicus, Corethron hystrix* |
| 10-12-2015 | 74.00 | 33.43 | 319 | 0 | 2 | 208 | *Halosphaera viridis* |
| 10-12-2015 | 74.00 | 33.43 | 319 | 50 | 2 | 61 | *Halosphaera viridis* |
| 21-11-2015 | 77.50 | 33.51 | 150 | 100 | 2 | 23 | *Prorocentrum* sp. |
| 13-04-2016 | 76.41 | 34.57 | 280 | 10 | 9 | 7 133 | *Phaeocystis pouchetii, Fragilariopsis oceanica, Chaetoceros socialis* |
| 13-04-2016 | 76.35 | 34.56 | 280 | 10 | 25 | 14 386 | *Phaeocystis pouchetii, Chaetoceros socialis* |
| 16-04-2016 | 78.21 | 43.93 | 270 | 10 | 41 | 23 868 | *Phaeocystis pouchetii, Prorocentrum balticum* |
| 16-04-2016 | 78.21 | 43.93 | 270 | 200 | 180 | 649 | *Oxytoxum caudatum* |
| 16-04-2016 | 78.21 | 43.93 | 270 | 270 | 27 | 117 | *Lessardia elongata* aff.*, Oxytoxum caudatum* |
| 17-04-2016 | 78.15 | 49.06 | 300 | 200 | 18 | 653 | *Lessardia elongata* aff.*, Pronoctiluca pelagica* |
| 20-04-2016 | 78.25 | 54.04 | 255 | 200 | 55 | 1 459 | *Phaeocystis pouchetii, Lessardia elongata* aff. |
| 04-12-2017 | 74.26 | 33.52 | 317 | 0 | 21 | 116 | *Coccolithus pelagicus, Oxytoxum caudatum* |
| 04-12-2017 | 74.26 | 33.52 | 317 | 20 | 29 | 74 | *Oxytoxum caudatum, Dicroerisma psilonereiella* |
| 04-12-2017 | 74.26 | 33.52 | 317 | 40 | 46 | 166 | *Oxytoxum caudatum, Coccolithus pelagicus, Corethron hystrix* |
| 04-12-2017 | 74.26 | 33.52 | 317 | 60 | 29 | 135 | *Oxytoxum caudatum, Dicroerisma psilonereiella, Ceratium fusus* |
| 04-12-2017 | 74.26 | 33.52 | 317 | 80 | 16 | 87 | *Oxytoxum caudatum, Coccolithus pelagicus, Corethron hystrix* |
| 04-12-2017 | 74.26 | 33.52 | 317 | 120 | 17 | 216 | *Coccolithus pelagicus, Dicroerisma psilonereiella, Ceratium fusus, Prorocentrum balticum* |
| 04-12-2017 | 74.26 | 33.52 | 317 | 150 | 13 | 254 | *Coccolithus pelagicus, Lessardia elongata* aff.*, Ceratium fusus* |
| 04-12-2017 | 74.26 | 33.52 | 317 | 200 | 12 | 223 | *Coccolithus pelagicus, Protoperidinium depressum, Ceratium fusus, Lessardia elongata* aff. |
| 04-12-2017 | 74.00 | 33.50 | 325 | 0 | 9 | 126 | *Coccolithus pelagicus, Ceratium fusus* |
| 04-12-2017 | 74.00 | 33.50 | 325 | 20 | 2 | 93 | *Coccolithus pelagicus, Ceratium fusus* |
| 04-12-2017 | 74.00 | 33.50 | 325 | 40 | 2 | 72 | *Coccolithus pelagicus, Ceratium fusus, Protoperidinium depressum* |
| 04-12-2017 | 74.00 | 33.50 | 325 | 60 | 4 | 114 | *Coccolithus pelagicus, Ceratium fusus* |
| 04-12-2017 | 74.00 | 33.50 | 325 | 80 | 8 | 109 | *Ceratium fusus, Prorocentrum balticum* |
| 04-12-2017 | 74.00 | 33.50 | 325 | 300 | 9 | 173 | *Coccolithus pelagicus, Lessardia elongata* aff.*, Prorocentrum balticum* |
| 05-12-2017 | 72.50 | 33.51 | 288 | 0 | 33 | 159 | *Oxytoxum caudatum, Dicroerisma psilonereiella* |
| 05-12-2017 | 72.50 | 33.51 | 288 | 20 | 47 | 145 | *Oxytoxum caudatum, Coccolithus pelagicus* |
| 21-06-2019 | 73.50 | 33.50 | 285 | 200 | 2 | 2 925 | *Chaetoceros socialis* |
| ***Podolampas palmipes* Stein** | | | | | | | |
| 10-11-2013 | 70.02 | 33.53 | 154 | 100 | 2 | 66 | *Dicroerisma psilonereiella* |
| 10-11-2013 | 70.50 | 33.48 | 250 | 65-0 | * | - | *Ceratium tripos, Ceratium fusus, Ceratium longipes, Halosphaera viridis* |
| 06-04-2016 | 70.00 | 33.51 | 140 | 120-50 | * | - | *Thalassiosira hyalina aff., Nitzschia grunowii* |
| 04-12-2017 | 74.26 | 33.52 | 317 | 100 | 1 | 104 | *Coccolithus pelagicus, Pronoctiluca pelagica, Prorocentrum balticum, Dicroerisma psilonereiella* |
| 05-12-2017 | 72.50 | 33.51 | 288 | 0 | 1 | 159 | *Oxytoxum caudatum, Dicroerisma psilonereiella* |
| 05-12-2017 | 72.50 | 33.51 | 288 | 20 | 1 | 145 | *Oxytoxum caudatum, Coccolithus pelagicus* |
| 25-01-2018 | 69.07 | 33.22 | 81 | 3 | 2 | 47 | *Melosira juergensii, Aulacoseira granulata, Ceratium fusus* |
| ***Proboscia indica* Hernandez-Becerril** | | | | | | | |
| 06-04-2016 | 70.00 | 33.51 | 140 | 50-0 | * | - | *Chaetoceros socialis, Chaetoceros furcellatus, Chaetoceros diadema, Thalassiosira gravida-antarctica, Thalassiosira hyalina aff.,* |
| 06-04-2016 | 70.00 | 33.51 | 140 | 120-50 | * | - | *Thalassiosira hyalina aff., Nitzschia grunowii* |
| ***Protoperidinium brochii* Balech** | | | | | | | |
| 10-11-2013 | 70.50 | 33.48 | 250 | 170-100 | * | - | *Protoperidinium pyriforme f.breve, Halosphaera viridis, Dinophysis rotundata* |
| 11-11-2013 | 71.50 | 33.51 | 278 | 240-155 | * | - | *Dinophysis rotundata, Dinophysis norvegica, Protoperidinium pyriforme f.breve, Ceratium arcticum* |
| ***Protoperidinium laticeps* Balech** | | | | | | | |
| 07-06-2014 | 72.45 | 23.20 | 301 | 14 | 5 | 1 136 | *Prorocentrum* sp.*, Nitzschia seriata* |
| 07-06-2014 | 72.45 | 23.20 | 301 | 301 | 2 | 66 | *Thalassionema nitzschioides* |
| 07-06-2014 | 72.03 | 24.07 | 284 | 9 | 14 | 1 710 | *Prorocentrum* sp.*, Protoperidinium pyriforme, Protoperidinium depressum* |
| 08-06-2014 | 74.00 | 33.50 | 319 | 0 | 39 | 26 895 | *Chaetoceros* sp.*, Chaetoceros laciniosus, Chaetoceros diadema* aff.*, Attheya longicornis* |
| 08-06-2014 | 74.00 | 33.50 | 319 | 3 | 13 | 79 645 | *Chaetoceros* sp.*, Chaetoceros diadema* aff.*, Attheya longicornis* |
| 08-06-2014 | 73.50 | 33.50 | 280 | 0 | 41 | 15 796 | *Chaetoceros* sp.*, Chaetoceros contortus* aff.*, Nitzschia delicatissima, Chaetoceros diadema* |
| 08-06-2014 | 73.50 | 33.50 | 280 | 21 | 4 | 13 011 | *Chaetoceros* sp. |
| 08-06-2014 | 73.00 | 33.50 | 214 | 5 | 98 | 33 095 | *Chaetoceros* sp.*, Chaetoceros diadema, Chaetoceros contortus* aff.*, Attheya longicornis* |
| 09-06-2014 | 72.50 | 33.50 | 246 | 0 | 3 | 1 611 | *Chaetoceros borealis, Prorocentrum* sp. |
| 09-06-2014 | 72.50 | 33.50 | 246 | 8 | 11 | 2 664 | *Chaetoceros borealis, Protoperidinium arcticum, Prorocentrum* sp. |
| 20-06-2019 | 72.50 | 33.50 | 288 | 20 | 4 | 4 261 | *Chaetoceros concavicornis* |
| 21-06-2019 | 74.00 | 33.50 | 315 | 25 | 26 | 283 099 | *Attheya longicornis, Chaetoceros concavicornis* |
| 21-06-2019 | 74.25 | 33.50 | 316 | 0 | 9 | 41 436 | *Attheya longicornis, Chaetoceros concavicornis, Thalassiosira gravida-antarctica* |
| 21-06-2019 | 74.50 | 33.50 | 262 | 30 | 19 | 240 195 | *Attheya longicornis, Chaetoceros concavicornis, Chaetoceros diadema, Dinobryon balticum* |
| 21-06-2019 | 74.50 | 33.50 | 262 | 0 | 4 | 144 091 | *Attheya longicornis, Chaetoceros concavicornis, Chaetoceros diadema, Dinobryon balticum* |
| 21-06-2019 | 74.50 | 33.50 | 262 | 20 | 13 | 194 642 | *Attheya longicornis, Chaetoceros concavicornis, Chaetoceros diadema* |
| ***Protoperidinium thulesense* (Balech) Balech** | | | | | | | |
| 20-06-2019 | 72.50 | 33.50 | 288 | 0 | 3 | 15 992 | *Attheya longicornis, Chaetoceros concavicornis* |
| 21-06-2019 | 73.50 | 33.50 | 285 | 0 | 8 | 233 174 | *Attheya longicornis, Chaetoceros concavicornis* |
| 21-06-2019 | 74.50 | 33.50 | 262 | 30 | 4 | 240 195 | *Attheya longicornis, Chaetoceros concavicornis, Chaetoceros diadema, Dinobryon balticum* |
| 21-06-2019 | 75.00 | 33.50 | 143 | 0 | 1 | 13 460 | *Dinobryon balticum* |
| ***Pseudophalacroma nasutum* Jörgensen** | | | | | | | |
| 10-11-2013 | 70.02 | 33.53 | 154 | 121-69 | * | - | *Protoperidinium pyriforme* f.breve*, Protoperidinium curtipes, Halosphaera viridis* |
| ***Pyrophacus horologicum* Stein** | | | | | | | |
| 11-11-2013 | 72.50 | 33.48 | 287 | 250-60 | * | - | *Ceratium arcticum, Protoperidinium depressum, Dinophysis rotundata* |
| 12-11-2013 | 74.00 | 33.50 | 326 | 10 | 2 | 110 | *Oxytoxum caudatum, Coccolithus pelagicus* |
| 12-11-2013 | 74.00 | 33.50 | 326 | 25 | 1 | 119 | *Coccolithus pelagicus, Oxytoxum caudatum* |
| 12-11-2013 | 74.50 | 33.50 | 257 | 0 | 3 | 91 | *Aulacoseira granulata, Coccolithus pelagicus, Oxytoxum caudatum* |
| 12-11-2013 | 74.50 | 33.50 | 257 | 10 | 4 | 151 | *Oxytoxum caudatum* |
| 12-11-2013 | 74.50 | 33.50 | 257 | 50 | 2 | 139 | *Oxytoxum caudatum, Mesoporos perforatus* |
| 12-11-2013 | 74.50 | 33.50 | 257 | 230-70 | * | - | *Ceratium arcticum, Ceratium fusus, Chaetoceros decipiens, Protoperidinium depressum* |
| 13-11-2013 | 76.02 | 33.48 | 306 | 0 | 2 | 95 | Mesoporos perforatus |
| 13-11-2013 | 76.02 | 33.48 | 306 | 50 | 1 | 74 | *Mesoporos perforatus, Coccolithus pelagicus* |
| 13-11-2013 | 76.02 | 33.48 | 306 | 100 | 1 | 81 | *Mesoporos perforatus* |
| ***Spatulodinium pseudonoctiluca* Cachon et Cachon** | | | | | | | |
| 24-07-2015 | 69.54 | 33.03 | 78 | 0 | 5 | 10 000 000 | *Pontosphaera huxleyi* |

*Values Not applied due to method of net sampling
